# Effects of Resveratrol Supplementation in Patients with Non-Alcoholic Fatty Liver Disease—A Meta-Analysis

**DOI:** 10.3390/nu12082435

**Published:** 2020-08-13

**Authors:** Karolina Jakubczyk, Karolina Skonieczna-Żydecka, Justyna Kałduńska, Ewa Stachowska, Izabela Gutowska, Katarzyna Janda

**Affiliations:** 1 Department of Human Nutrition and Metabolomics, Pomeranian Medical University in Szczecin, 24 Broniewskiego Street, 71-460 Szczecin, Poland; karjak@pum.edu.pl (K.J.); justynakaldunska@wp.pl (J.K.); ewast@pum.edu.pl (E.S.); katarzyna.janda@pum.edu.pl (K.J.); 2 Department of Medical Chemistry, Pomeranian Medical University, Powstancow Wlkp Street 72, 71-460 Szczecin, Poland; gutowska@pum.edu.pl

**Keywords:** Non-alcoholic fatty liver disease (NAFLD), resveratrol, meta-analysis

## Abstract

Non-alcoholic fatty liver disease (NAFLD) is regarded as one of the most common liver pathologies in many societies. Resveratrol, as a phenolic compound with powerful antioxidant and anti-inflammatory properties exerting positive effects on the lipid profile and lipid accumulation and also on insulin resistance, appears to be an effective, natural, and safe complementary treatment option in NAFLD therapy. This meta-analysis was undertaken to evaluate the effects of resveratrol supplementation in NAFLD patients. To this end, scientific databases PubMed/Medline/Embase were searched up to 19 March 2020. We included seven randomized clinical trials (RCTs) with a total of 302 patients with NAFLD. In all the trials included in the analysis, resveratrol was administered daily over periods between 56 and 180 days in doses ranging from 500 mg to 3000 mg a day. The results of this meta-analysis reveal that resveratrol supplementation, irrespective of the dose or duration, did not affect the analyzed parameters (*p* < 0.05). The sole exception was an increase in alanine aminotransferase following the administration of resveratrol (*p* = 0.041). Currently available evidence is insufficient to confirm the efficacy of resveratrol in the management of NAFLD. Due to the inconsistencies between the existing scientific reports, a number of which found a positive effect on NAFLD-related parameters; further research in this area is needed.

## 1. Introduction

Non-alcoholic fatty liver disease (NAFLD) encompasses a range of disorders, from simple fatty liver (steatosis) without signs of liver cell injury, through complicated fatty liver disease (steatohepatitis) accompanied by a persistent inflammatory condition, to the development of cirrhosis [1,2,3,4]. It is currently the most prevalent chronic liver disease in overweight people, affecting up to one third in this patient group [1,2,4]. The onset and progression of NAFLD is a complex process involving numerous factors and mechanisms, such as e.g., dyslipidemia, insulin resistance, overweight and obesity, oxidative stress, arterial hypertension, inflammation, lipid metabolism disorders, dysbiosis, and genetic factors [1,2,4,5,6,7]. In the majority of patients, NAFLD is asymptomatic. When symptoms occur, they may include abdominal pain in the right upper quadrant, fatigue, malaise, enlarged liver and, to a lesser extent, enlarged spleen. Additionally, patients frequently present with slightly elevated serum levels of aspartate aminotransferase (AST) and alanine aminotransferase (ALT) [8,9]. These symptoms have been implicated as key risk factors for many metabolic disorders, including cardiovascular diseases, type 2 diabetes, lipid disorders, and NAFLD [10]. Research findings suggest that some natural substances may exert antioxidant and anti-inflammatory effects and successfully support NAFLD therapy [11,12,13].

Resveratrol (3,5,4’-trihydroxy-trans-stilbene) is a naturally-occurring phenolic stilbene [14,15]. The chemical structure of this compound is similar to synthetic estrogen diethylstilbestrol, and that is why it is classified as a phytoestrogen. It is formed via a condensation reaction between three molecules of malonyl-CoA and one molecule of 4-coumaroyl-CoA catalyzed by resveratrol synthase (RS) [14]. Resveratrol (RSV) is found in many plant species. Its sources include peanuts, legumes, pomegranate, and pine. Large quantities of RSV are also present in grapes and grapevine products, e.g., wine. The majority of supplements containing resveratrol are made from Japanese knotweed (*Polygonum cuspidatum*) [15].

As much as 75% of resveratrol is absorbed in the small intestine, and then transported via blood to the liver, where with the participation of cytochrome P450 it is bio-converted into its glucuronide form, trans-resveratrol. Small quantities are also metabolized to sulphates [14]. In the human body, resveratrol is rapidly cleared, hence its plasma levels are low. Resveratrol metabolites are excreted with bile and urine [14]. Resveratrol is a compound affecting the induction of many factors in the body, notably including sirtuins (Sirt1, Sirt3, Sirt4) proteins with enzymatic activity, which in turn regulate the activity of forkhead box-containing (FOX) proteins—a family of transcription factors which play an important role in regulating the expression of genes involved in cell growth, proliferation, differentiation, and longevity [16]. Resveratrol also stimulates apoptosis in adipocytes and slows down their formation, inhibits lipogenesis, activates fatty acid oxidation processes, and increases the rate of thermogenesis. The substance has powerful anti-inflammatory and antioxidant properties [3,16,17,18,19]. 

These mechanisms suggest that resveratrol supplementation may be effective in the prevention and management of NAFLD [13,20,21,22]. On the other hand, clinical trials have produced conflicting results as to the efficacy of resveratrol in the treatment of this disease [8,17,23,24,25,26,27]. That is why we have undertaken a comprehensive meta-analysis, aiming to verify whether resveratrol supplementation is beneficial for NAFLD patients. Our study is based on randomized clinical trials (RCT).

## 2. Materials and Methods 

### 2.1. Search Strategy and Inclusion Criteria

At least two independent authors (K.J., J.K., and K.S.-Z.) searched PubMed/Medline/Embase databases from their inception until 19/03/2020 without language restriction for randomized controlled trials (RCTs) comparing resveratrol supplementation to controls (placebo/no treatment) in NAFLD.

The following search term was used in PubMed: human AND (nonalcoholic fatty liver OR nafld (nonalcoholic fatty liver disease) OR non alcoholic fatty liver disease OR non alcoholic hepatosteatosis OR non alcoholic liver steatosis OR non-alcoholic fld OR non-alcoholic fatty liver OR non-alcoholic fatty liver disease OR non-alcoholic hepatic steatosis OR nonalcoholic fld OR nonalcoholic fatty liver OR nonalcoholic fatty liver disease OR nonalcoholic hepatic steatosis OR nonalcoholic hepatosteatosis OR nonalcoholic liver steatosis) AND (resveratrol 3,4`,5 stilbenetriol OR 3,4`,5 trihydroxystilbene OR 5 (4 hydroxystyryl) benzene 1,3 diol OR resveratrol OR srt 501 OR srt501 OR trans resveratrol OR trans-resveratrol) AND (alanine aminotransferase OR gpt OR alanine aminotransferase OR alanine 2 oxoglutarate aminotransferase OR alanine 2 oxoisovalerate aminotransferase OR alanine alpha ketoglutarate transaminase OR alanine alpha oxoglutarate transaminase OR alanine amino transferase OR alanine aminotransferase OR alanine transaminase OR alanine transpeptidase OR alanine 2 oxoglutarate aminotransferase OR e.c. 2.6.1.2 OR glutamate alanine transaminase OR glutamate pyruvate aminotransferase OR glutamate pyruvate transaminase OR glutamate pyruvate transaminase OR glutamic alanine aminotransferase OR glutamic pyruvate transaminase OR glutamic pyruvic aminotransferase OR glutamic pyruvic transaminase OR glutamo pyruvic transaminase OR l alanine 2 oxoglutarate aminotransferase OR l alanine 2 oxoglutarate aminotransferase OR aspartate aminotransferase OR 1 aspartate 2 oxoglutarate aminotransferase OR 1 aspartate 2 oxoglutarate aminotransferase OR got OR aminotransferase, aspartate OR aspartate amino transferase OR aspartate aminotransferase OR aspartate aminotransferases OR aspartate transaminase OR aspartic aminotransferase OR aspartic transaminase OR e.c. 2.6.1.1 OR glutamate aspartate transaminase OR glutamate oxalacetate transaminase OR glutamate oxalacetic transaminase OR glutamate oxalate transaminase OR glutamate oxalic transaminase OR glutamate oxaloacetate aminotransferase OR glutamate oxaloacetate transaminase OR glutamate oxaloacetic acid transaminase OR glutamate oxalacetate transaminase OR glutamic aspartic aminotransferase OR glutamic aspartic transaminase OR glutamic oxal acetat transaminase OR glutamic oxalacetic acid transaminase OR glutamic oxalacetic transaminase OR glutamic oxalacetic transferase OR glutamic oxalic transaminase OR glutamic oxaloacetic acid transaminase OR glutamic oxaloacetic aminotransferase OR glutamic oxaloacetic transaminase OR glutamine oxalacetic transaminase OR glutaminic oxaloacetic transaminase OR l aspartate 2 oxoglutarate transaminase OR l aspartate 2 oxoglutarate aminotransferase OR l aspartate aminotransferase OR l aspartate 2 oxoglutarate aminotransferase OR l aspartate 2 oxoglutarate transaminase OR l aspartate 2 oxoglutarate aminotransferase OR levo aspartate aminotransferase OR transaminase a OR gamma glutamyl transferase blood level OR gamma glutamyl transferase blood level OR gamma glutamyl transpeptidase blood level OR gamma glutamyltransferase/blood level OR glutamyl transpeptidase blood level OR plasma glutamyl transpeptidase OR serum 4 glutamyl transpeptidase OR serum gamma glutamyl transpeptidase OR serum gamma glutamyltranspeptidase OR serum gamma gt OR serum glutamyl transpeptidase OR serum glutamyltranspeptidase OR bilirubin blood level OR bilirubin blood level OR bilirubin serum level OR bilirubin, serum OR blood bilirubin OR plasma bilirubin OR serum bilirubin OR cholesterol OR 3 hydroxy 5 cholestene OR 3beta hydroxy 5 cholestene OR 3beta hydroxycholest 5 ene OR 5 cholesten 3beta ol OR beta cholesterol OR cholest 5 en 3beta ol OR cholest 5 ene 3 ol OR cholesterin OR cholesterine OR cholesterol OR cholesterol release OR dithiol OR nsc 8798 OR high density lipoprotein cholesterol OR hdl cholesterol OR cholesterol, hdl OR high density lipoprotein cholesterol OR lipoproteins, hdl cholesterol OR low density lipoprotein cholesterol OR ldl cholesterol OR cholesterol, ldl OR lipoproteins, ldl cholesterol OR low density lipoprotein cholesterol OR triacylglycerol OR acylglycerol, tri OR fatty acid triglyceride OR triacylglyceride OR triacylglycerol OR triglyceride OR triglycerides OR triglyceride). 

In Embase the string was as follows: ‘human’ AND (‘nonalcoholic fatty liver’/exp OR ‘nafld (nonalcoholic fatty liver disease)’ OR ‘non alcoholic fatty liver disease’ OR ‘non alcoholic hepatosteatosis’ OR ‘non alcoholic liver steatosis’ OR ‘non-alcoholic fld’ OR ‘non-alcoholic fatty liver’ OR ‘non-alcoholic fatty liver disease’ OR ‘non-alcoholic hepatic steatosis’ OR ‘nonalcoholic fld’ OR ‘nonalcoholic fatty liver’ OR ‘nonalcoholic fatty liver disease’ OR ‘nonalcoholic hepatic steatosis’ OR ‘nonalcoholic hepatosteatosis’ OR ‘nonalcoholic liver steatosis’) AND (‘resveratrol’/exp OR ‘3, 4`, 5 stilbenetriol’ OR ‘3, 4`, 5 trihydroxystilbene’ OR ‘5 (4 hydroxystyryl) benzene 1, 3 diol’ OR ‘resveratrol’ OR ‘srt 501’ OR ‘srt501’ OR ‘trans resveratrol’ OR ‘trans-resveratrol’) AND (‘alanine aminotransferase’/exp OR ‘gpt’ OR ‘alanin aminotransferase’ OR ‘alanine 2 oxoglutarate aminotransferase’ OR ‘alanine 2 oxoisovalerate aminotransferase’ OR ‘alanine alpha ketoglutarate transaminase’ OR ‘alanine alpha oxoglutarate transaminase’ OR ‘alanine amino transferase’ OR ‘alanine aminotransferase’ OR ‘alanine transaminase’ OR ‘alanine transpeptidase’ OR ‘alanine:2 oxoglutarate aminotransferase’ OR ‘e.c. 2.6.1.2’ OR ‘glutamate alanine transaminase’ OR ‘glutamate pyruvate aminotransferase’ OR ‘glutamate pyruvate transaminase’ OR ‘glutamate pyruvatetransaminase’ OR ‘glutamic alanine aminotransferase’ OR ‘glutamic pyruvate transaminase’ OR ‘glutamic pyruvic aminotransferase’ OR ‘glutamic pyruvic transaminase’ OR ‘glutamopyruvic transaminase’ OR ‘l alanine 2 oxoglutarate aminotransferase’ OR ‘l alanine:2 oxoglutarate aminotransferase’ OR ‘aspartate aminotransferase’/exp OR ‘1 aspartate 2 oxoglutarate aminotransferase’ OR ‘1 aspartate:2 oxoglutarate aminotransferase’ OR ‘got’ OR ‘aminotransferase, aspartate’ OR ‘aspartate amino transferase’ OR ‘aspartate aminotransferase’ OR ‘aspartate aminotransferases’ OR ‘aspartate transaminase’ OR ‘aspartic aminotransferase’ OR ‘aspartic transaminase’ OR ‘e.c. 2.6.1.1’ OR ‘glutamate aspartate transaminase’ OR ‘glutamate oxalacetate transaminase’ OR ‘glutamate oxalacetic transaminase’ OR ‘glutamate oxalate transaminase’ OR ‘glutamate oxalic transaminase’ OR ‘glutamate oxaloacetate aminotransferase’ OR ‘glutamate oxaloacetate transaminase’ OR ‘glutamate oxaloacetic acid transaminase’ OR ‘glutamatoxalacetate transaminase’ OR ‘glutamic aspartic aminotransferase’ OR ‘glutamic aspartic transaminase’ OR ‘glutamic oxal acetatic transaminase’ OR ‘glutamic oxalacetic acid transaminase’ OR ‘glutamic oxalacetic transaminase’ OR ‘glutamic oxalacetic transferase’ OR ‘glutamic oxalic transaminase’ OR ‘glutamic oxaloacetic acid transaminase’ OR ‘glutamic oxaloacetic aminotransferase’ OR ‘glutamic oxaloacetic transaminase’ OR ‘glutamine oxaloacetic transaminase’ OR ‘glutaminic oxalacetic transaminase’ OR ‘l asparate 2 oxoglutarate transaminase’ OR ‘l aspartate 2 oxoglutarate aminotransferase’ OR ‘l aspartate aminotransferase’ OR ‘l aspartate:2 oxoglutarate aminotransferase’ OR ‘l aspartate:2 oxoglutarate transaminase’ OR ‘l aspatate:2 oxoglutarate aminotransferase’ OR ‘levo aspartate aminotransferase’ OR ‘transaminase a’ OR ‘gamma glutamyl transferase blood level’/exp OR ‘gamma glutamyl transferase blood level’ OR ‘gamma glutamyl transpeptidase blood level’ OR ‘gamma glutamyltransferase blood level’ OR ‘glutamyl transpeptidase blood level’ OR ‘plasma glutamyl transpeptidase’ OR ‘serum 4 glutamyl transpeptidase’ OR ‘serum gamma glutamyl transpeptidase’ OR ‘serum gamma glutamyltranspeptidase’ OR ‘serum gamma gt’ OR ‘serum glutamyl transpeptidase’ OR ‘serum glutamyltranspeptidase’ OR ‘bilirubin blood level’/exp OR ‘bilirubin blood level’ OR ‘bilirubin serum level’ OR ‘bilirubin, serum’ OR ‘blood bilirubin’ OR ‘plasma bilirubin’ OR ‘serum bilirubin’ OR ‘cholesterol’/exp OR ‘3 hydroxy 5 cholestene’ OR ‘3beta hydroxy 5 cholestene’ OR ‘3 beta hydroxycholest 5 ene’ OR ‘5 cholesten 3beta ol’ OR ‘beta cholesterol’ OR ‘cholest 5 en 3beta ol’ OR ‘cholest 5 ene 3 ol’ OR ‘cholesterin’ OR ‘cholesterine’ OR ‘cholesterol’ OR ‘cholesterol release’ OR ‘dythol’ OR ‘nsc 8798’ OR ‘high density lipoprotein cholesterol’/exp OR ‘hdl cholesterol’ OR ‘cholesterol, hdl’ OR ‘high density lipoprotein cholesterol’ OR ‘lipoproteins, hdl cholesterol’ OR ‘low density lipoprotein cholesterol’/exp OR ‘ldl cholesterol’ OR ‘cholesterol, ldl’ OR ‘lipoproteins, ldl cholesterol’ OR ‘low density lipoprotein cholesterol’ OR ‘triacylglycerol’/exp OR ‘acylglycerol, tri’ OR ‘fatty acid triglyceride’ OR ‘triacyl glyceride’ OR ‘triacylglycerol’ OR ‘triglyceride’ OR ‘triglycerides’ OR ‘tryglyceride’). The electronic search was supplemented by a manual review of reference lists from eligible publications and relevant reviews. 

Inclusion criteria were: (i) randomized controlled trial, (ii) confirmed diagnosis of NAFLD, (iii) treatment with resveratrol at any dose, (iv) randomization to resveratrol vs. placebo/no treatment, (v) available meta-analyzable change score/endpoint (preferred) data on NAFLD-associated parameters We excluded studies evaluating RSV intervention compared to other products containing bioactive substances.

### 2.2. Data Abstraction 

Data on study design, risk of bias [28], patient, illness, and treatment characteristics from each study were independently extracted in accordance with the Preferred Reporting Items for Systematic Reviews and Meta-Analyses (PRISMA) standard by two independent investigators (K.J. and J.K.). Whenever data were missing for the review, authors were contacted for additional information via emails twice, two weeks apart. Inconsistencies were resolved by consensus and the corresponding author was involved. Data from charts and figures were extracted by means of WebPlotDigitizer software (https://automeris.io/WebPlotDigitizer/).

### 2.3. Outcomes

The co-primary outcomes that were extracted from each study were the NAFLD-related parameters associated with the putative mechanism of resveratrol action, among them: aspartate aminotransferase (AST), alanine aminotransferase (ALT), glutamate alanine transaminase, liver fat content, low-density lipoprotein (LDL), high-density lipoprotein (HDL), total cholesterol (TC), triacylglycerol (TAG), gamma-glutamyl transferase (GGTP), hepatic steatosis, body mass index (BMI), body weight, insulin, glucose level, and Homeostatic Model Assessment of Insulin Resistance (HOMA IR).

### 2.4. Data Synthesis and Statistical Analysis

We conducted a random-effects [29] meta-analysis of outcomes for which ≥2 studies contributed data, using Comprehensive Meta-Analysis V3 (http://www.meta-analysis.com). We explored study heterogeneity using the chi-square test of homogeneity, with *p* < 0.05 indicating significant heterogeneity. All analyses were two-tailed with alpha = 0.05.

Group differences in continuous outcomes were analyzed as the pooled standardized mean difference (SMD) in either endpoint scores (preferred) or change from baseline to endpoint using observed cases (OC). 

We inspected funnel plots and used Egger’s regression test [30] and the Duval and Tweedie’s trim and fill method [31] to quantify whether publication bias could have influenced the results.

## 3. Results

### 3.1. Search Results

**Figure 1:**
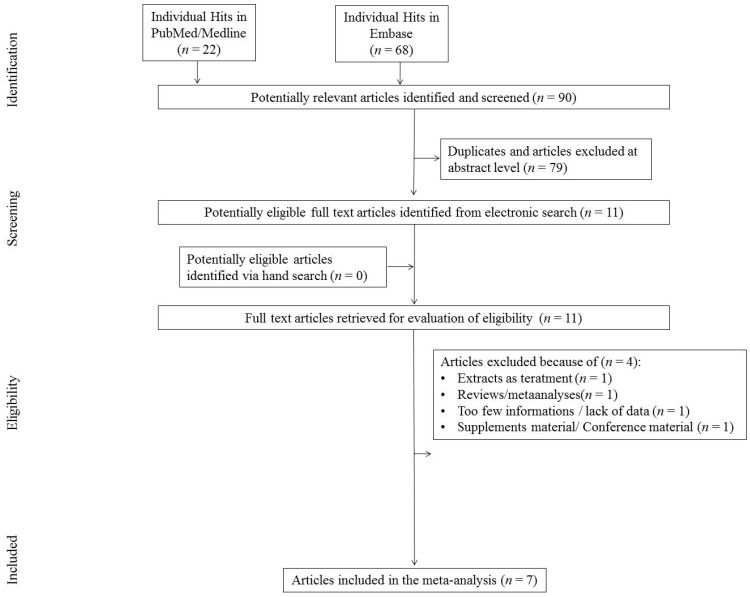
Flowchart.

The initial search yielded 90 hits. 79 studies were excluded for being duplicates and/or after evaluation on the title/abstract level. There were 11 additional articles identified via hand search. During the next step 11 full-text articles were included into present study to be full-text reviewed. Of those, four were excluded as they did not fit inclusion criteria. Primary reasons for exclusion were: review (*n* = 1), lack of necessary data on outcomes (*n* = 1), supplementary material (*n* = 1), and studies with extract as treatment agent (*n* = 1; Figure 1), finally yielding seven studies that were included in the meta-analysis to be thoroughly reviewed. 

### 3.2. Study, Patient, and Treatment Characteristics

Altogether, seven studies comprising *n* = 302 (a total of 358 were randomized and 302 analyzed) participants were included. All patients were diagnosed with NAFLD. The age of studied persons was around 40 years and ranged between 39.25 and 48.15 years. The mean number of males was 224 (74.17%) and BMI, if provided, ranged between 25.3 ± 2.11 and 32.1 ± 3.1 kg/m^2^. The studies we included aimed to analyze the impact of RSV supplementation on different NAFLD related parameters, e.g., liver enzymes, lipid and insulin levels, insulin resistance, and others. In all studies the intervention was RSV that was ingested by patients every day at a dose between 500–3000 mg/day. The mean study duration was 94.57 ± 39.27 days (range = 56–180 days). In all interventions, RSV capsules were administered. Details are placed in Table 1.

### 3.3. Risk of Bias (ROB)

The mean number of low risk-of-bias assessments in all studies included in the meta-analysis was 4.14 (median = 5) [9,16,19,32,33,34,35]. There were four [16,32,33,34] studies with the score “5” and three [9,19,35] studies with “3” low ROB assessments. Details on ROB evaluation are given in Table 2.

### 3.4. The Impact of Resveratrol on NAFLD-Related Outcomes

**Figure 2:**
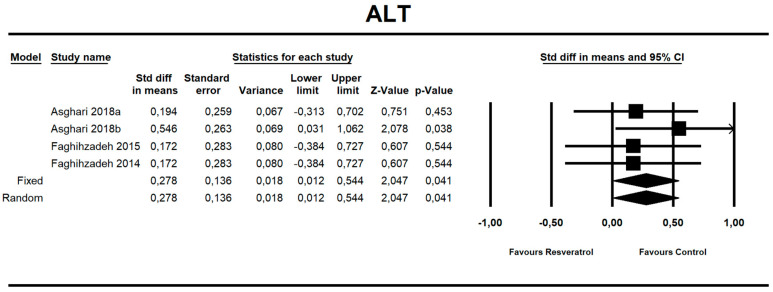
Effect size, standardized mean difference, for ALT in persons taking RSV *vs*. controls (endpoint data). Q = 1.427, df(Q) = 3, *p* = 0.699, *I*-squared = 0.0.

Using random-effects meta-analysis, we observed that RVS ingestion significantly affected the ALT level (Standardized differencein means (SDM): 0.278 with a 95% confidence interval of 0.012 to 0.544; *z* = 2.047, *p* = 0.041; Figure S1). Surprisingly, the RSV ingestion resulted in increased level of the enzyme. Using random-effects meta-analysis, we observed that RVS ingestion did not significantly affect other co-primary outcomes evaluated in present study as shown in Table 3. Forest plots (Figure 2 and Figures S1–S12), additional parameters related to NAFLD that were not included in the meta-analysis (Table S1), and Baseline parameters (Table S2) are placed in supplementary data.

Egger’s tests showed no significant publication bias in the current meta-analysis (for all study outcomes *p* > 0.05; Figures S13–S22). 

## 4. Discussion

Non-alcoholic fatty liver disease (NAFLD) is regarded as one of the most common liver pathologies prevalent in many societies. NAFLD may progress into non-alcoholic steatohepatitis (NASH), and then to liver cirrhosis, the main cause of hepatocellular carcinoma. Consequently, its prevention and therapy figure importantly in the hepatology of those disease entities [2,4,36].

The multiple pathways of NAFLD pathogenesis mean that there are no less than a few potential treatment approaches, which are being studied around the globe. Resveratrol, as a polyphenol with powerful antioxidant properties inhibiting lipid accumulation, improving insulin sensitivity, and reducing triglyceride levels in serum, appears to be an effective, natural, and safe complementary treatment option in NAFLD therapy, particularly arising as a result of obesity and/or insulin resistance [2,36,37,38]. The beneficial effects of resveratrol may also be due to its ability to modulate hepatocellular apoptosis processes, slow down the progression of fatty liver disease, and its anti-inflammatory properties [2,37,38]. This would suggest that resveratrol therapy helps reduce the risks related to the onset and development of NAFLD.

The total number of participants included in this meta-analysis was 302 individuals, with women outnumbering the men. In all the studies included in the analysis, resveratrol was administered daily over periods between 56 and 180 days with doses ranging from 500 mg to 3000 mg a day. In all the trials, resveratrol was administered in the form of capsules. The results of the studies included in the meta-analysis are nevertheless still unclear. Four out of the RCTs included reported a beneficial effect of resveratrol on some parameters related to non-alcoholic fatty liver disease [9,19,34,35]. Conversely, the results of the other three trials indicate that resveratrol failed to produce a long-term therapeutic benefit in NAFLD [16,32,33]. In the study carried out by Asghari et al., a significant reduction of weight (by 1.1%) and BMI was observed following resveratrol administration (600 mg, 84 days) compared to the placebo group (*p* < 0.05). ALT, AST, and lipid profile parameters did not change significantly in the group receiving resveratrol (all *p* > 0.05). No significant changes were observed in the grade of liver steatosis, glycemic parameters in serum, high-density lipoprotein cholesterol, and sirtuin-1 levels (*p* > 0.05) [16]. The next study, likewise, showed no changes in the serum levels of liver enzymes (ALT, AST, GGT—gamma-glutamyl transferase, and ALP—alkaline phosphatase; *p* > 0.05) [32]. Administering 3000 mg resveratrol for eight weeks did not reduce insulin resistance, steatosis grade, or abdominal fat distribution compared to baseline. Additionally, no change was observed in plasma lipids, while the levels of alanine and aspartate aminotransferases increased significantly compared to the placebo group [33]. In turn, Chen et al. demonstrated that resveratrol supplementation (600 mg, 90 days) significantly decreased aspartate aminotransferase, glucose, and low-density lipoprotein [34]. Faghihzadeh et al. in both studies demonstrated that resveratrol supplementation significantly suppressed ALT activity and liver steatosis compared to the placebo group (*p* < 0.05). No other changes were observed with regard to anthropometric measurements, insulin resistance markers, lipid profile, or blood pressure [35]. Heebøll et al. observed no consistent therapeutic effects of resveratrol supplementation in alleviating clinical or histological NAFLD, but found a small improvement in lipid parameters (total cholesterol, HDL, and LDL). Additionally, adverse effects were reported, including fever and bicytopenia [9]. In light of such controversial findings, this meta-analysis was undertaken to provide reliable evidence as to the efficacy of resveratrol supplementation in NAFLD. 

It is crucial to emphasize that diet itself might be a factor influencing the results we obtained. In three studies, the authors recommended a specific diet to diminish the probability of worsening the NAFLD course due to inappropriate nutrition [16,19,35]. In one study, nutritional education of patients has been adopted [32]. Patients form Asghari et al. study were subjected to a caloric deficit (500 to 1000 kcal/d) based on their body weight. The proportions of carbohydrates, fats, and protein were 53%, 30%, and 17% respectively. Faghihzadeg et al. [19,35] utilized a similar diet providing total fat, ≤30%; total energy value, SFA, 10%; MUFA, 15%; PUFA, 5%; protein, 15–18%; carbohydrates, 52–55%; cholesterol dietary, <300 mg/d; and 20–30 g of fiber/d. In addition, they were advised to exercise at least for 30 min, three times per week. Furthermore, the authors demonstrated that modifications in diet contributed to improving anthropometric measurements (weight, body mass index, waist circumference), hepatic enzymes, and grade of steatosis (*p* < 0.05). However, these changes were observed in both groups: experimental and control [19]. While trials investigating resveratrol therapy in patients with non-alcoholic fatty liver disease produce dissimilar, often ambiguous effects, in this meta-analysis we were not able to prove any beneficial effects of resveratrol supplementation. The results of this meta-analysis demonstrate that resveratrol supplementation, irrespective of the dose or duration, did not produce benefits to patients with non-alcoholic fatty liver disease compared to the placebo—as evaluated in a random-effects model, which is consistent with other reports [8,9,16,19,23,32,33].

Liver enzymes (AST, ALT) are regarded as indicators of NAFLD progression, hence it is so important to reduce their levels in the course of therapy [39,40]. What is more, NAFLD patients frequently present slightly elevated levels of these parameters [8,9]. In the present study, it was demonstrated that the decline in aspartate aminotransferase following resveratrol treatment in NAFLD patients was not statistically significant. Alanine aminotransferase was an exception, as resveratrol therapy boosted its activity compared to placebo (*p* = 0.41). Two RCT studies confirmed a significant increase of both liver enzymes following resveratrol treatment [16,33]. Please note, however, that these changes were also observed in the placebo group. Furthermore, in the majority of pooled studies, the experimental group presented with much higher enzyme levels at baseline than the control group. On the other hand, two studies showed a decline in the activity of liver enzymes in the group receiving resveratrol or in both groups [9,34]. Faghihzadeh et al. also reported a decline in alanine aminotransferase [19,35]. In the meta-analysis conducted by Zhang et al., [23] resveratrol was demonstrated not to affect the activity of liver enzymes. In turn, a study focusing on patients with NAFLD showed that a six-month therapy with resveratrol supplementation contributed to decreasing the levels of hepatic enzymes [37]. The effects of resveratrol on liver enzymes appears to be inconsistent. Nevertheless, most studies report its positive effects. It appears to be of paramount importance to take into account liver enzyme parameters in the allocation of patients into groups, as it may significantly affect results.

In this meta-analysis, we observed no clear relationship between resveratrol supplementation and change in anthropometric measurements (body weight, BMI, WC), which is consistent with some of the studies included in our analysis [9,16,32,33,34]. This finding is also consistent with previous meta-analyses, encompassing 158 patients [8] and 156 patients, each including four RCTs [23]. It is worth noting that two RCTs [19,35] reported a significant decline in these parameters. In one of the trials, BMI and WC scores were down in both groups, although there were no significant differences between the two groups [35], while in the other trial a significant decline across all the anthropometric parameters was observed in both the supplemented and placebo groups [19]. These reports are important as obesity is regarded as one of the main risk factors for liver cirrhosis. Earlier studies using rodent models showed resveratrol was capable of inhibiting the progression of NAFLD by contributing to weight loss and bringing down blood sugar levels and LDL cholesterol [41,42,43].

For patients with NAFLD, glucose metabolism is an important parameter. Our meta-analysis showed no positive effects of resveratrol on glucose metabolism, which is consistent with all the published RCTs. The study by Chen et al. is an exception, reporting a significant decline in blood sugar levels [42]. The authors linked this effect to a lower dosage and longer duration of supplementation than in the study by Chachay [33,34]. Nevertheless, other studies, with a similar dose and duration of intervention, failed to produce the same effect [16,19,32,35]. An improvement in parameters related to insulin resistance was also shown in a study not included in this analysis, where patients with NAFLD were given a resveratrol supplement over a six-month observation period [37].

Resveratrol has been reported to reduce lipid accumulation, improve insulin sensitivity and bring down triglyceride levels in blood serum [2,36,38]. We did not observe statistically significant differences in lipid profile parameters following resveratrol supplementation. On the other hand, in the meta-analysis conducted by Zhang et al., pooling 156 participants of four independent research studies, it was demonstrated that resveratrol supplementation increased total and LDL cholesterol levels [23]. The pool of studies included in our meta-analysis includes that by Faghihzadeh et al. [35], where a significant increase in total cholesterol was noted after resveratrol administration (*p* < 0.05). In the study by Asghari et al. [16], an increase in TAG, TC, and LDL after resveratrol administration was observed, contrary to the observations made by Chen et al., where LDL and TC levels declined compared to the placebo [34]. Please note that the dose, duration of supplementation and the number of participants in both trials were similar. Yet, the patients’ BMI scores in the study by Chen et al. were in the 20–30 kg/m^2^ range, with fasting blood glucose <7.8 mmol/L, while in the study by Asghari et al. the BMI range was 25–35 kg/m^2^, and fasting blood glucose was not taken into account. The trials also provided for different exclusion criteria, potentially contributing to the differences in observations [16,34].

Based on the obtained results, no obvious correlation was found between resveratrol supplementation and changes in systolic blood pressure (SBP) and diastolic blood pressure (DBP). RCT studies, as well as earlier meta-analyses, did not report improvements in these parameters, either [8,23]. Yet, Timmers et al. reported that resveratrol supplementation in obese patients at 150 mg a day over a period of 30 days brought down SBP by 5 mmHg. These inconsistent results may be attributed to dissimilar doses of resveratrol administered in the respective studies, and primarily to the comorbidity of non-alcoholic fatty liver disease, which is frequently accompanied by hypertension, an additional risk factor for the development of the disease [2,44]. 

Several limitations of this meta-analysis need to be considered. These include (i) the relatively small sample size; (ii) heterogeneous inclusion criteria in individual RCTs, particularly with respect to the levels of liver enzymes (ASP, ALT); BMI scores in the experimental and control group; (iii) differing doses and durations of resveratrol supplementation; and (iv) different inconsistent exclusion criteria. We also did not stratify studies to dietary patterns. In fact, all these limitations may be interpreted as factors undermining resveratrol efficacy. Thus, to clearly demonstrate the efficacy of such an intervention, it is necessary to undertake research with appropriate group allocation criteria with reference to the above parameters. Moreover, the critical problems derived from NAFLD are liver fibrosis and carcinogenesis thus these outcomes are still awaiting to be assessed under RSV supplementation.

We conducted a thorough assessment of the risk of bias in each of the studies and in general found the quality of the included samples to be high. We assume that research protocols differed widely, and this variable may remain as the so-called small effect size. We are also aware of the potential bias in the review process, as we may have overlooked certain studies which did not explicitly focus on resveratrol intervention in NAFLD patients but may have reported relevant findings.

## 5. Conclusions

In summary, our meta-analysis shows that there is no sufficient evidence for the efficacy of resveratrol supplementation in patients with non-alcoholic fatty liver disease. Resveratrol was not found to exert a significant effect in terms of reducing anthropometric parameters, lipid profile, glucose metabolism, or arterial pressure. On the other hand, an increase in alanine aminotransferase was observed after resveratrol intake, which appears to be controversial. Hence, further research is necessary, in light of the inconsistencies in the existing scientific reports, where beneficial effects on parameters important in NAFLD were repeatedly observed. Furthermore, it seems to be imperative to account for the levels of hepatic enzymes when allocating patients into groups, determining dose or duration of supplementation, as it may potentially affect the obtained results. 

## Figures and Tables

**Table 1:** Study characteristics.

No.	Study Description				Intervention			Sample Characteristics				
	Reference/Year/Country/Sponsorship	Blinding/ Crossover (Y/N)	Focus on	ROB	Form/RSVtype	RSV Dose (mg/day)	Duration of RSV Administration (days)/ Comparator	*N* Total Randomized/ Analyzed	Age Years (mean ± SD)	Males (*n*/%)	BMI Baseline (kg/m^2^): RSV Group (Mean ± SD)	BMI Baseline (kg/m^2^): Control Group (mean ± SD)
1	Asghari et al./ 2018a/Iran/ Non-industry	SB/N	Liver enzymes, lipid and insulin levels, insulin resistance	5	Capsules, pure trans-RSV	600	84/placebo	60/60	39.53 ± 6.72	35/58.33	30.78 (±3.1)	30.41 (±3.39)
2	Asghari et al./ 2018b/Iran/ Non-industry	DB/N	Liver enzymes, levels	5	Capsules, pure trans-RSV	600	84/placebo	60/60	39.25 ± 26.53	40/66.66	ND	ND
3	Chachay et al./ 2014/China/Non-industry	DB/N	Liver enzymes, lipid insulin, bilirubin, IL-6, CRP, TNF-α levels, DBP, SBP, insulin resistance	5	Capsules	3000	56/placebo	20/20	48.15 ± 11.73	20/100	31.8 (30.2–37)	31.2 (27.4–39.3)
4	Chen et al./ 2015/Australia/Non-industry	DB/N	Insulin resistance, glucose and lipid metabolism	5	Capsules (from natural products)	600	90/placebo	60/60	44.30 ± 10.5	42/70	25.3 (±2.11)	26.2 (±3.08)
5	Faghihzadeh et al./ 2015/Iran/Non-industry	DB/N	Liver enzymes, lipid insulin, glucose, bilirubin, levels, DBP, SBP, insulin resistance	3	Capsules	500	84/placebo	50/48	41.16 ± 9.81	35/70	28.35 (±3.49)	28.75 (±3.5)
6	Faghihzadeh et al./ 2014/Iran/Non-industry	DB/N	Liver enzymes, lipid, bilirubin, IL-6, CRP, TNF-α levels	3	Capsules, pure trans-RSV	500	84/placebo	50/48	41.16 ± 9.81	35/70	28.35 (±3.49)	28.75 (±33.5
7	Heebøll et al./ 2016/Denmark/Non-industry	DB/N	Liver enzymes, lipid insulin, glucose, bilirubin, TNF-α levels, DBP, SBP, insulin resistance	3	Capsules, pure trans-RSV	1500	180/placebo	28/26	43.2 (22–67) PBO 43,5 (21–69) ^^	17/65.38	32,1 (±3.1)	32 (±5.4)

DB, double blind; SB, single blind; N, no; Y, yes; ROB, risk of bias; SD, standard deviation; DBP, diastolic blood pressure; SBP, systolic blood pressure; RSV, resveratrol; BMI, body mass index; IL-6, Interleukin 6; CRP, C-reactive protein; TNF-α, tumor necrosis factor α; PBO, placebo; ND, No data; ^^, quadriles.

**Table 2:** Risk of bias.

No.	Reference/Year/Country/Sponsorship	Random Sequence Generation (Selection Bias)	Allocation Concealment (Selection Bias)	Blinding of Participants and Personnel (Performance Bias)	Blinding of Outcome Assessment (Detection Bias)	Incomplete Outcome Data Addressed (Attrition Bias)	Selective Reporting (Reporting Bias)	Other Bias	No. Of Low Assessments
1	Asghari et al./2018a/Iran/ Non-industry	L	L	L	?	L	L	?	5
2	Asghari et al./2018b/Iran/ Non-industry	L	L	L	?	L	L	?	5
3	Chachay et al./2014/China/Non-industry	L	L	L	?	L	L	?	5
4	Chen et al./2015/Australia/Non-industry	L	L	L	?	L	L	?	5
5	Faghihzadeh et al./2015/Iran/Non-industry	?	?	L	?	L	L	?	3
6	Faghihzadeh et al./2014/Iran/Non-industry	?	?	L	?	L	L	?	3
7	Heebøll et al./2016/Denmark/Non-industry	H	H	L	?	L	L	?	3

L, low risk of bias; H, high risk of bias; ?, unclear risk of bias.

**Table 3:** The effects sizes, SMD, for co-primary outcomes analyzed in present meta-analysis.

Outcome	SMD	95%CI	Z	*p*
AST	0.052	−0.202, 0.307	0.404	0.686
Body weight	−0.061	−0.334, 0.212	−0.438	0.661
BMI	−0.076	−0.364, 0.212	−0.518	0.604
WC	−0.075	−0.385. 0.236	−0.471	0.638
Glucose	−0.184	−0.585, 0.218	−0.897	0.370
Insulin	−0.178	−0.948, 0.593	−0.452	0.651
TC	−0.053	−0.401, 0.296	−0.297	0.767
TAG	−0.095	−0.470, 0.280	−0.496	0.620
LDL	0.225	−0.122, 0.571	1.270	0.204
HDL	−0.184	−0.559, 0.191	−0.959	0.337
SBP	−0.035	−0.379, 0.310	−0.197	0.844
DBP	0.118	−0.345, 0.580	0.498	0.618

## Supplementary Materials

The following are available online at https://www.mdpi.com/2072-6643/12/8/2435/s1, Figure S1. Effect size, standardized mean difference, for AST in persons taking RSV vs. controls (endpoint data). Q = 3.938, df(Q) = 4, *p* = 0.415, I-squared = 0.0. Figure S2. Effect size, standardized mean difference, for BMI in persons taking RSV vs. controls (endpoint data). Q = 0.180, df(Q) = 3, *p* = 0.981, I-squared = 0.0; Figure S3. Effect size, standardized mean difference, for body weight in persons taking RSV vs. controls (endpoint data). Q = 0.546, df(Q) = 4, *p* = 0.969, I-squared = 0.0; Figure S4. Effect size, standardized mean difference, for WC in persons taking RSV vs. controls (endpoint data). Q = 0.484, df(Q) = 2, *p* =0.785, I-squared = 0.0; Figure S5. Effect size, standardized mean difference, for glucose in persons taking RSV vs. controls (endpoint data). Q = 0.450, df(Q) = 2, *p* = 0.798, I-squared = 0.0; Figure S6. Effect size, standardized mean difference, for insulin in persons taking RSV vs. controls (endpoint data). Q = 0.00, df(Q) = 0, *p* = 1.000, I-squared = 0.0; Figure S7. Effect size, standardized mean difference, for TC in persons taking RSV vs. controls (endpoint data). Q = 7.185, df(Q) = 2, *p* = 0.028, I-squared = 72.163; Figure S8. Effect size, standardized mean difference, for TAG in persons taking RSV vs. controls (endpoint data). Q = 0.942, df(Q) = 1, *p* = 0.332, I-squared = 0.0; Figure S9. Effect size, standardized mean difference, for LDL in persons taking RSV vs. controls (endpoint data). Q = 2.392, df(Q) = 2, *p* = 0.302, I-squared = 16.396; Figure S10. Effect size, standardized mean difference, for HDL in persons taking RSV vs. controls (endpoint data). Q = 0.532, df(Q) = 1, *p* = 0.466, I-squared = 0.0; Figure S11. Effect size, standardized mean difference, for SBP in persons taking RSV vs. controls (endpoint data). Q = 4.638, df(Q) = 3, *p* = 0.200, I-squared = 35.317; Figure S12. Effect size, standardized mean difference, for DBP in persons taking RSV vs. controls (endpoint data). Q = 0.007, df(Q) = 2, *p* = 0.996, I-squared = 0.0; Figure S13. Funnel plot for the effect of resveratrol on the level of ALT in patients with NAFLD in present meta-analysis; Figure S14. Funnel plot for the effect of resveratrol on the level of AST in patients with NAFLD in present meta-analysis; Figure S15. Funnel plot for the effect of resveratrol on the body weight in patients with NAFLD in present meta-analysis; Figure S16. Funnel plot for the effect of resveratrol on the BMI in patients with NAFLD in present meta-analysis; Figure S17. Funnel plot for the effect of resveratrol on the WC in patients with NAFLD in present meta-analysis; Figure S18. Funnel plot for the effect of resveratrol on the level of glucose in patients with NAFLD in present meta-analysis; Figure S19. Funnel plot for the effect of resveratrol on the level of TC in patients with NAFLD in present meta-analysis; Figure S20. Funnel plot for the effect of resveratrol on the level of LDL in patients with NAFLD in present meta-analysis; Figure S21. Funnel plot for the effect of resveratrol on the level of SBP in patients with NAFLD in present meta-analysis; Figure S22. Funnel plot for the effect of resveratrol on the level of DBP in patients with NAFLD in present meta-analysis. Table S1. Additional parameters related to NAFLD that were not included in the meta-analysis, Table S2. Baseline parameters related to NAFLD.
