# Supplementary material for: Effects of Resveratrol Supplementation in Patients with Non-Alcoholic Fatty Liver Disease—A Meta-Analysis
Source: Nutrients. 2020 Aug 13;12(8):2435. doi: 10.3390/nu12082435 (PMC7469003; doi:10.3390/nu12082435)
Supplement: Supplementary file 1 [file nutrients-12-02435-s001.zip › Supplementary_Figure.docx]

**Supplementary Figure**


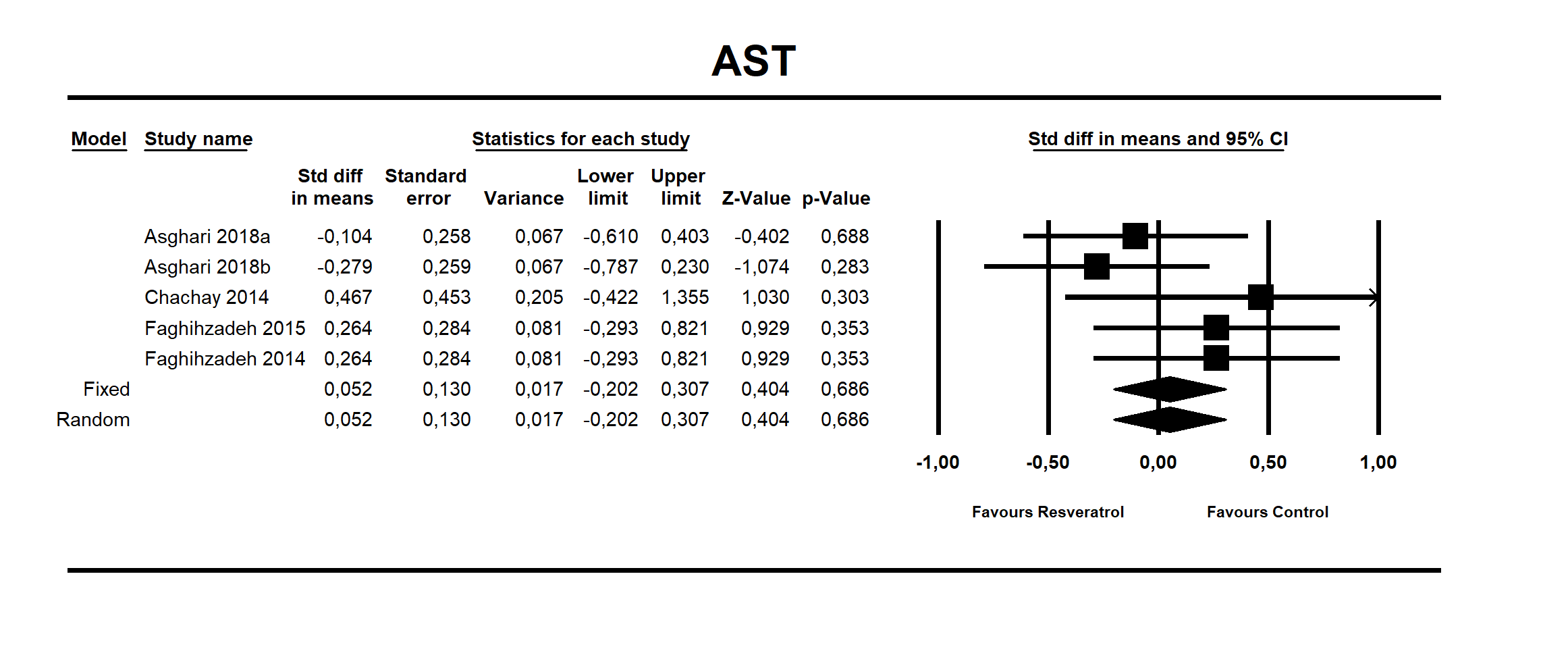


**Figure 1.** An effect size, standardized mean difference, for AST in persons taking RSV vs. controls (endpoint data). Q = 3.938, df(Q) = 4, *p* =0.415, I-squared = 0.0.


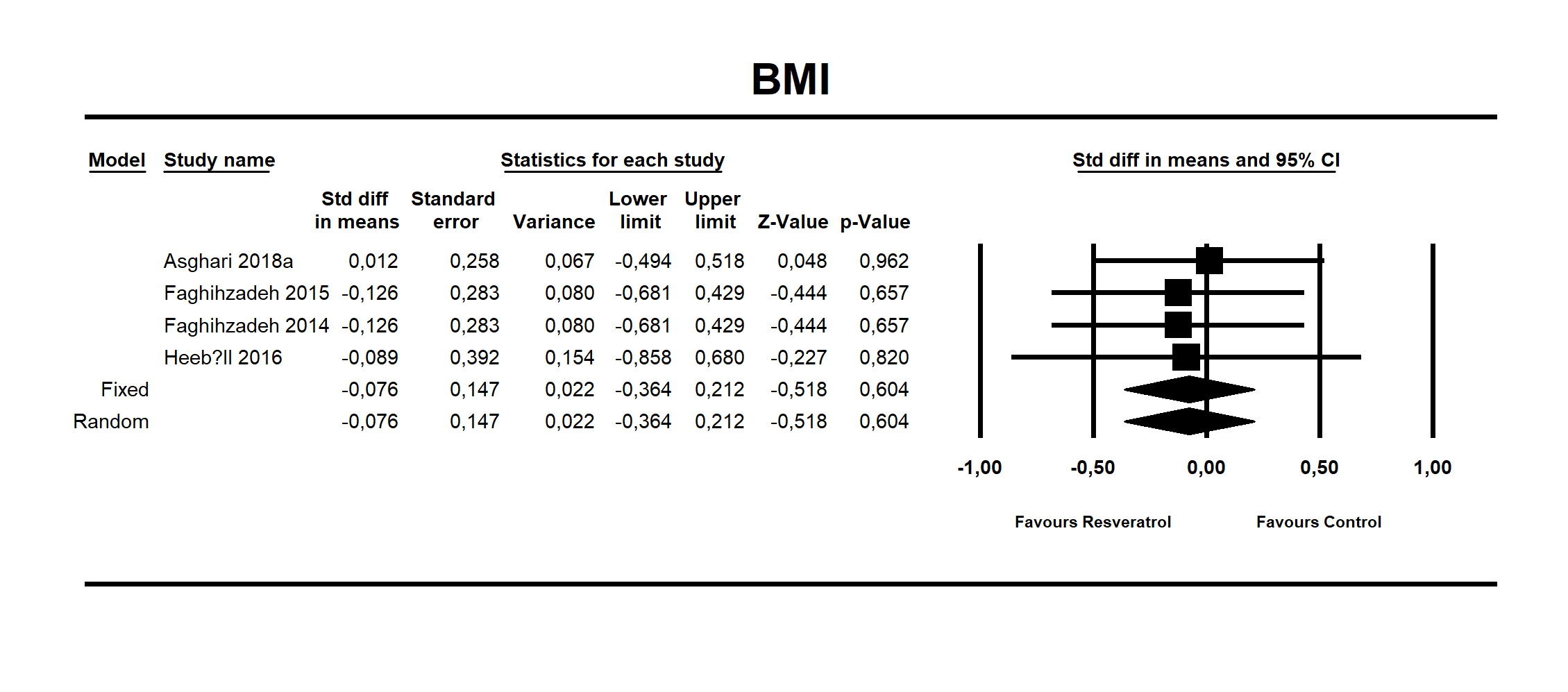


**Figure 2.** An effect size, standardized mean difference, for BMI in persons taking RSV vs. controls (endpoint data). Q = 0.180, df(Q) = 3, *p* =0.981, I-squared = 0.0.


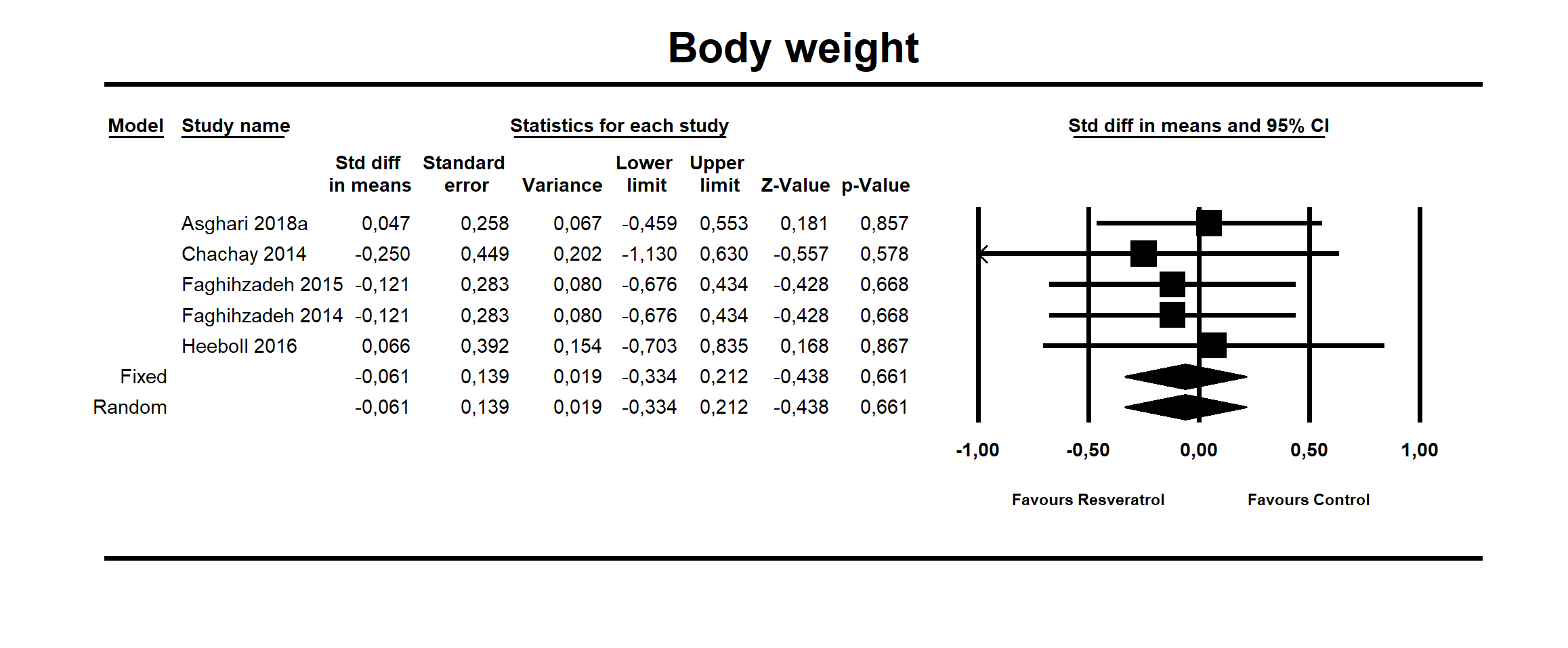


**Figure 3.** An effect size, standardized mean difference, for body weight in persons taking RSV vs. controls (endpoint data). Q = 0.546, df(Q) = 4, *p* =0.969, I-squared = 0.0.


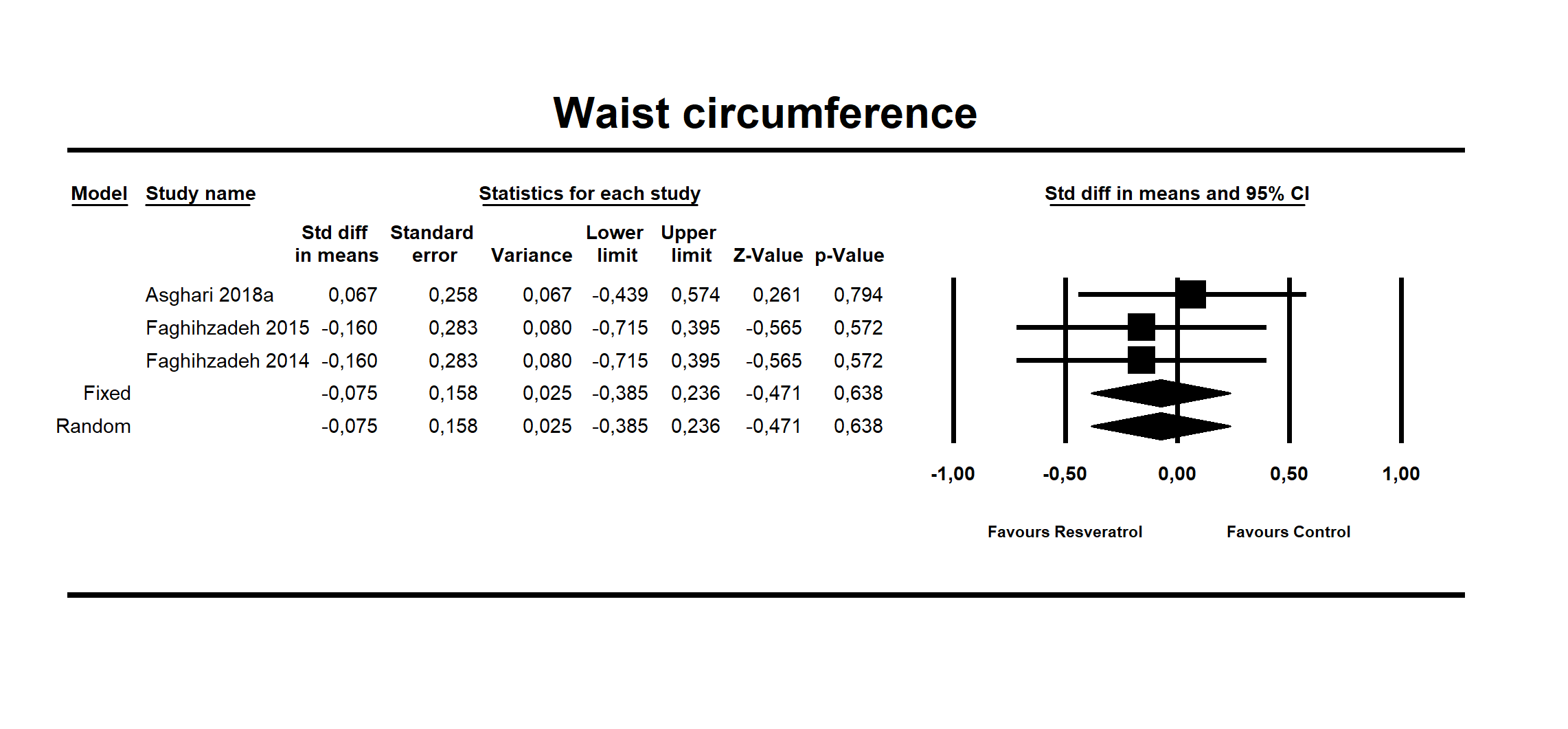


**Figure 4.** An effect size, standardized mean difference, for WC in persons taking RSV vs. controls (endpoint data). Q = 0.484, df(Q) = 2, *p* =0.785, I-squared = 0.0.


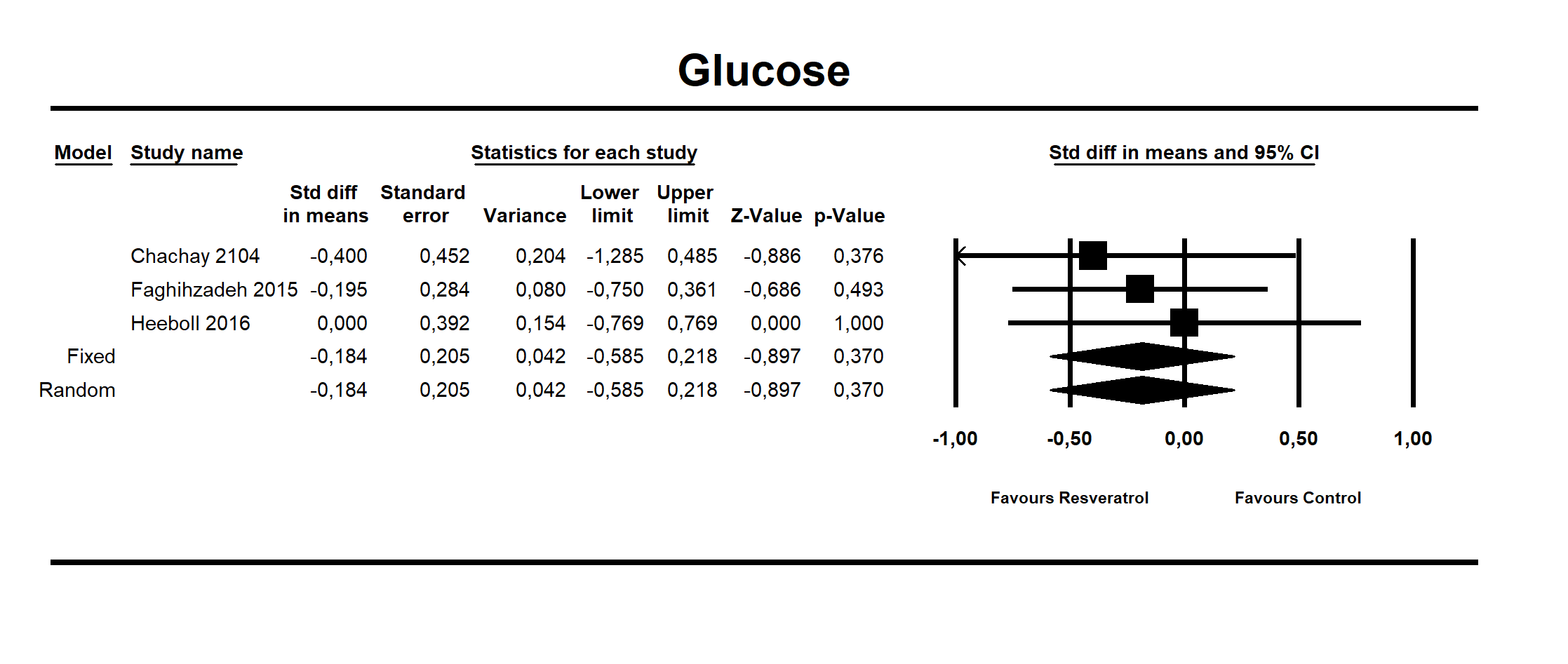


**Figure 5.** An effect size, standardized mean difference, for glucose in persons taking RSV vs. controls (endpoint data). Q = 0.450, df(Q) = 2, *p* =0.798, I-squared = 0.0.


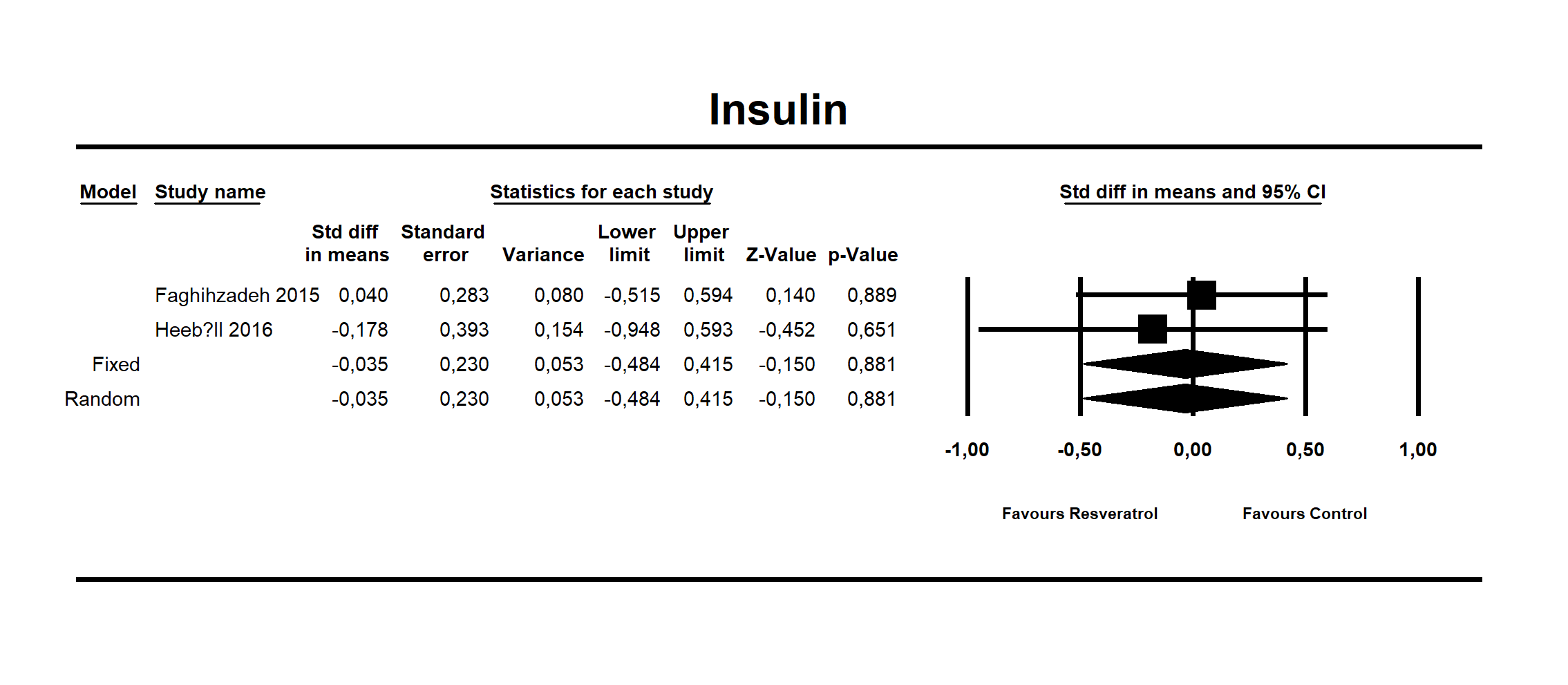


**Figure 6.** An effect size, standardized mean difference, for insulin in persons taking RSV vs. controls (endpoint data). Q = 0.00, df(Q) = 0, *p* =1.000, I-squared = 0.0.


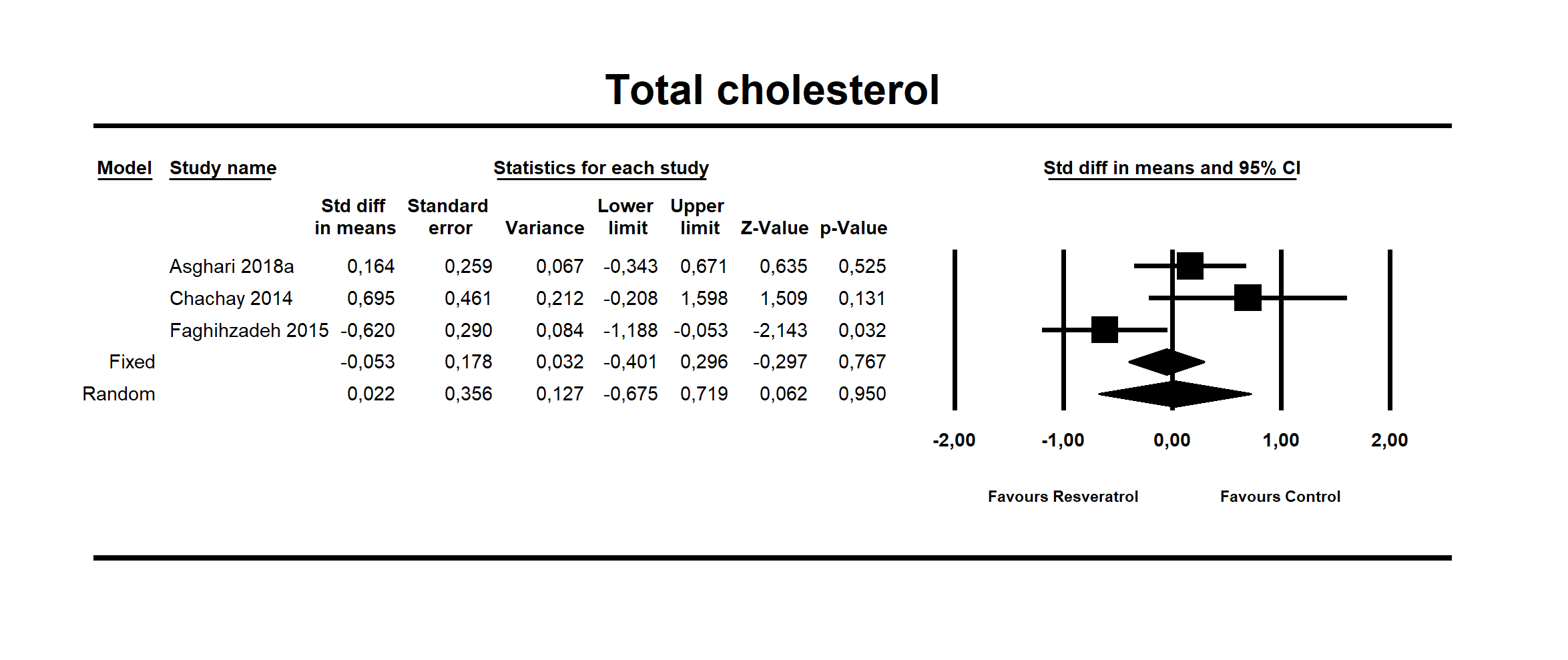


**Figure 7.** An effect size, standardized mean difference, for TC in persons taking RSV vs. controls (endpoint data). Q = 7.185, df(Q) = 2, *p* =0.028, I-squared = 72.163.


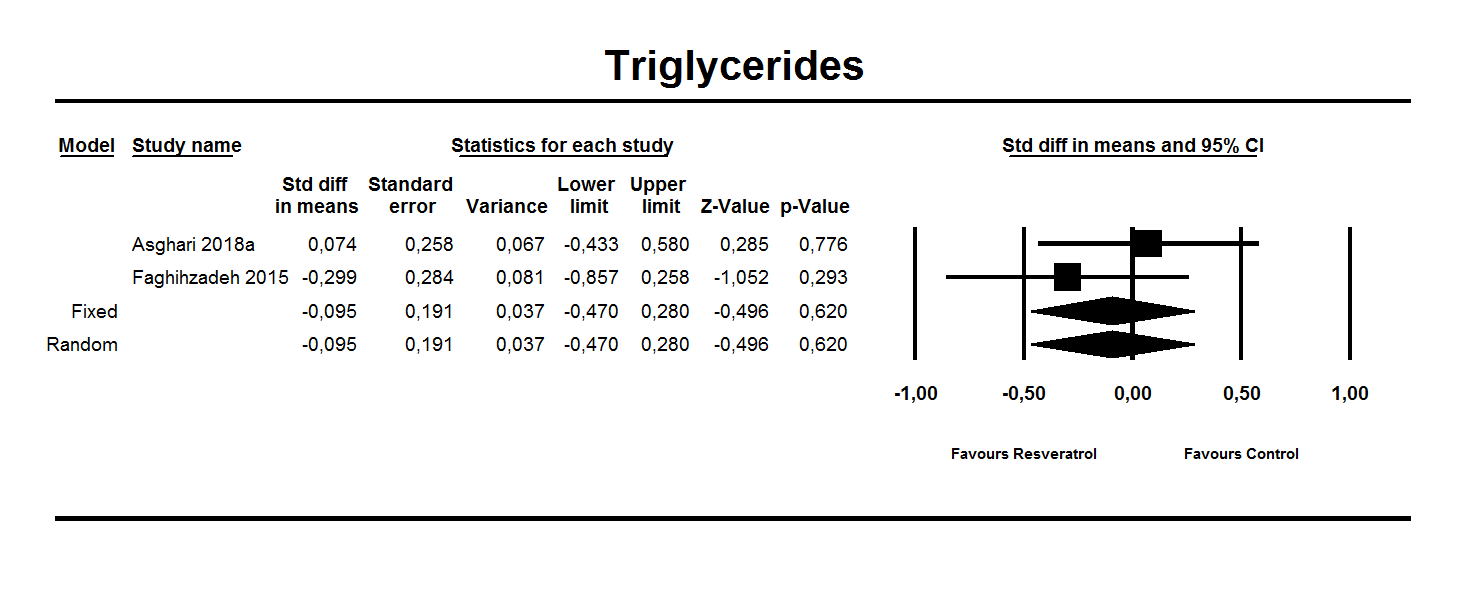


**Figure 8.** An effect size, standardized mean difference, for TAG in persons taking RSV vs. controls (endpoint data). Q = 0.942, df(Q) = 1, *p* =0.332, I-squared = 0.0.


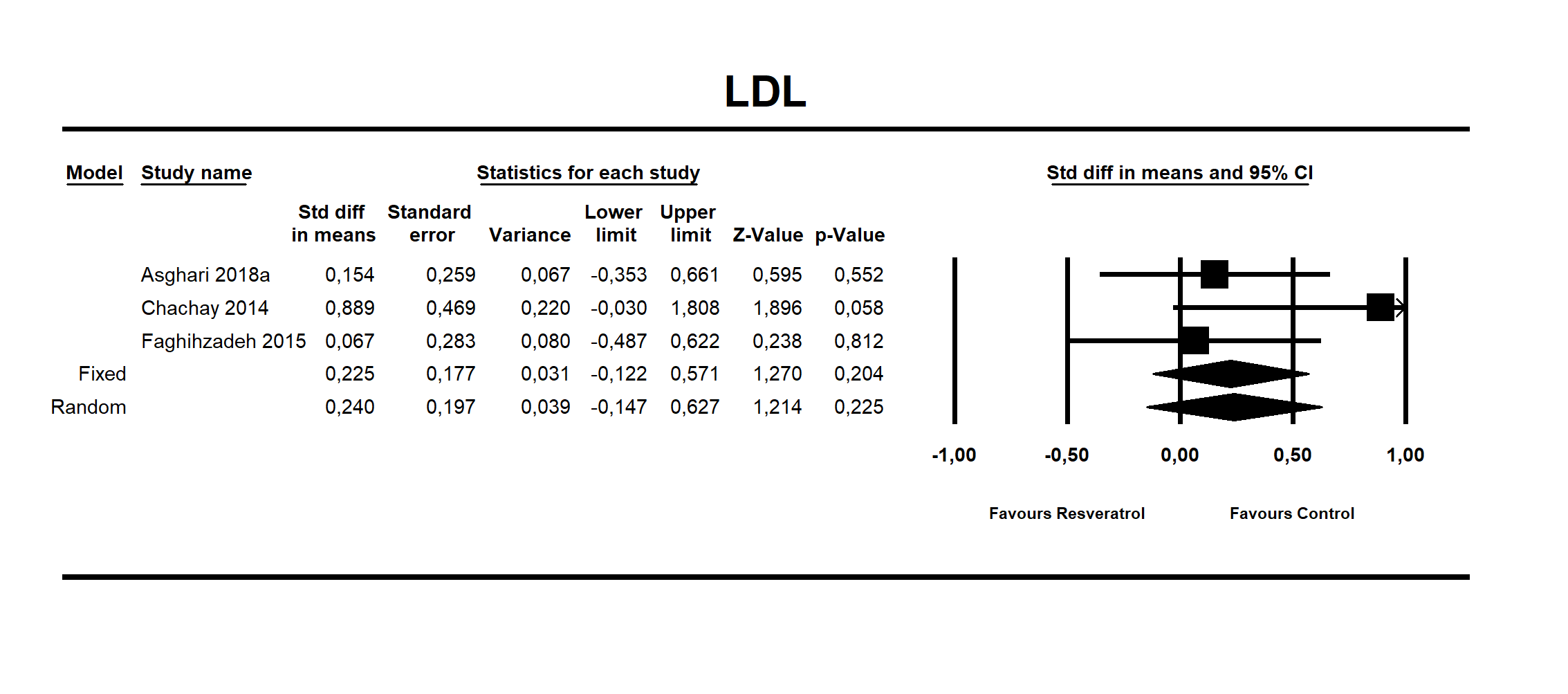


**Figure 9.** An effect size, standardized mean difference, for LDL in persons taking RSV vs. controls (endpoint data). Q = 2.392, df(Q) = 2, *p* =0.302, I-squared = 16.396.


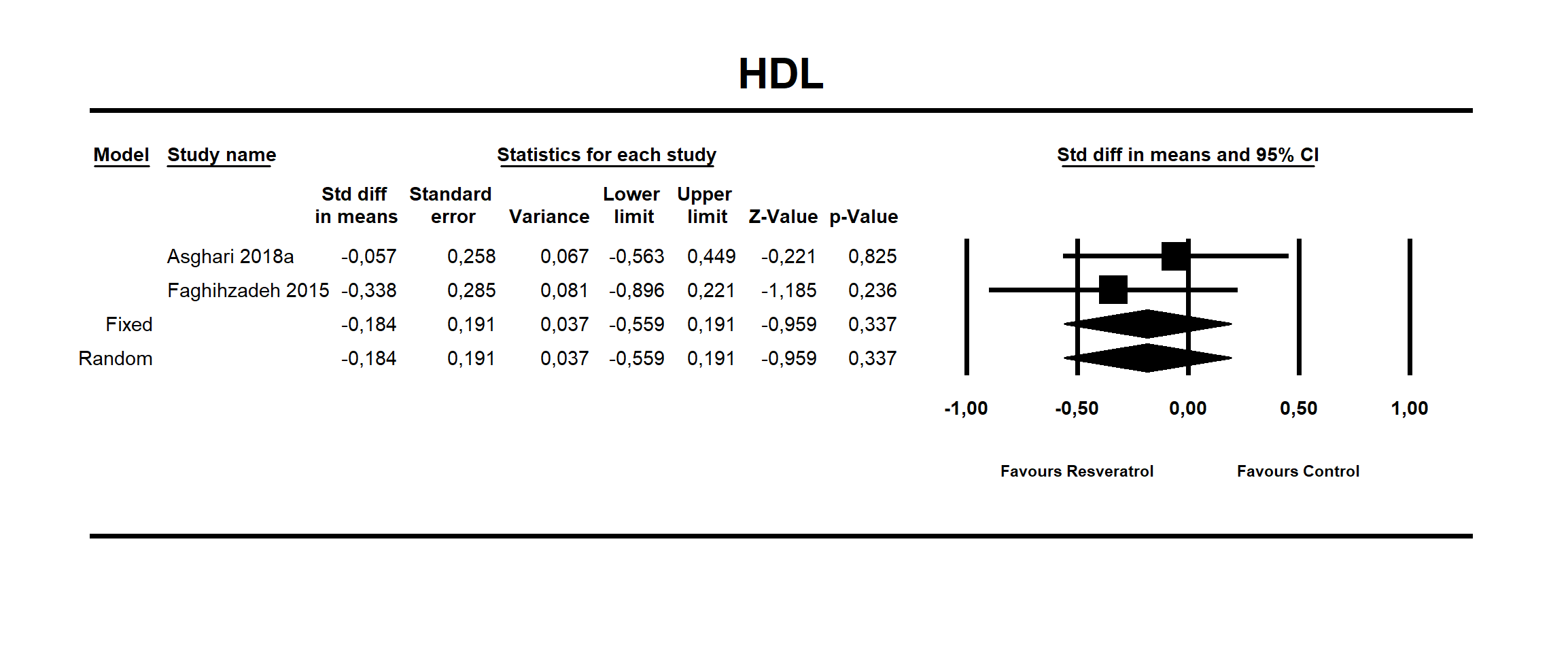


**Figure 10.** An effect size, standardized mean difference, for HDL in persons taking RSV vs. controls (endpoint data). Q = 0.532, df(Q) = 1, *p* =0.466, I-squared = 0.0.


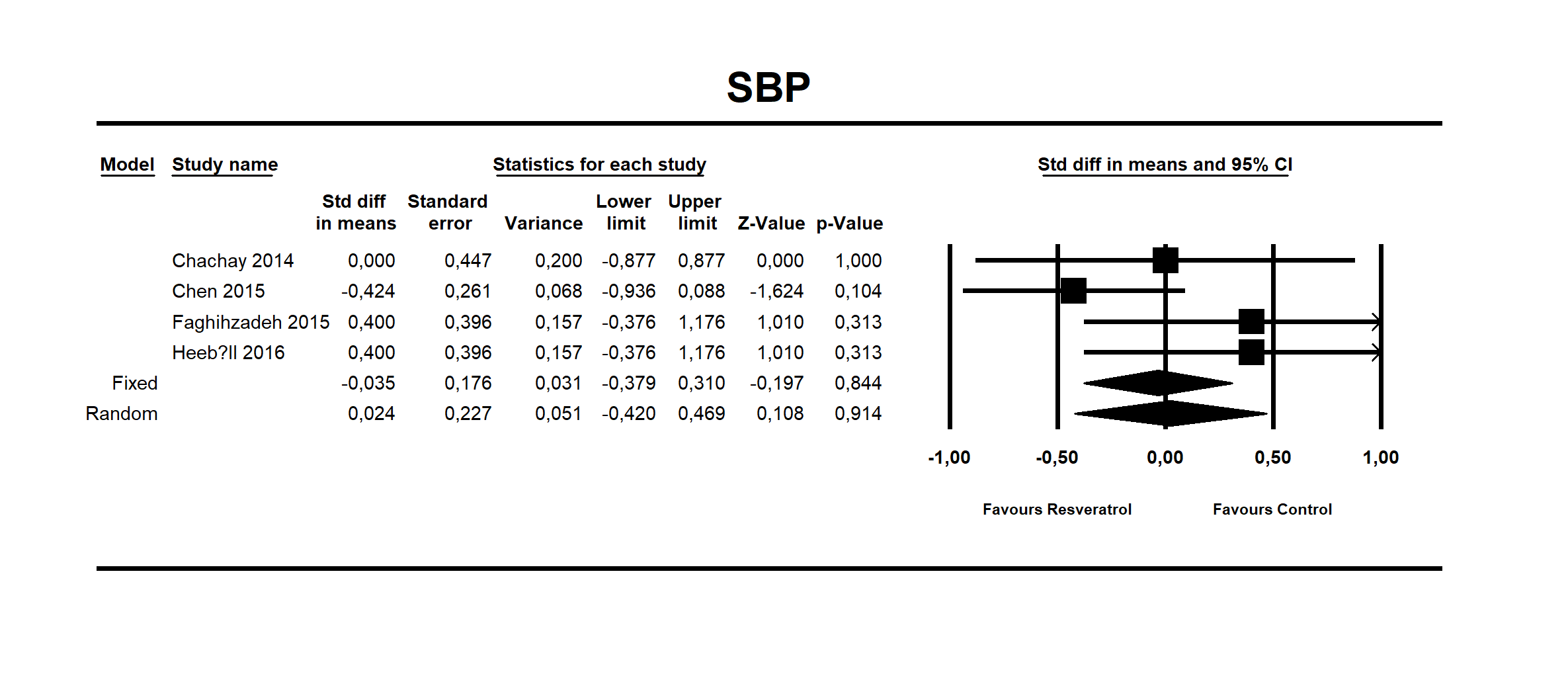


**Figure 11.** An effect size, standardized mean difference, for SBP in persons taking RSV vs. controls (endpoint data). Q = 4.638, df(Q) = 3, *p* =0.200, I-squared = 35.317.

**
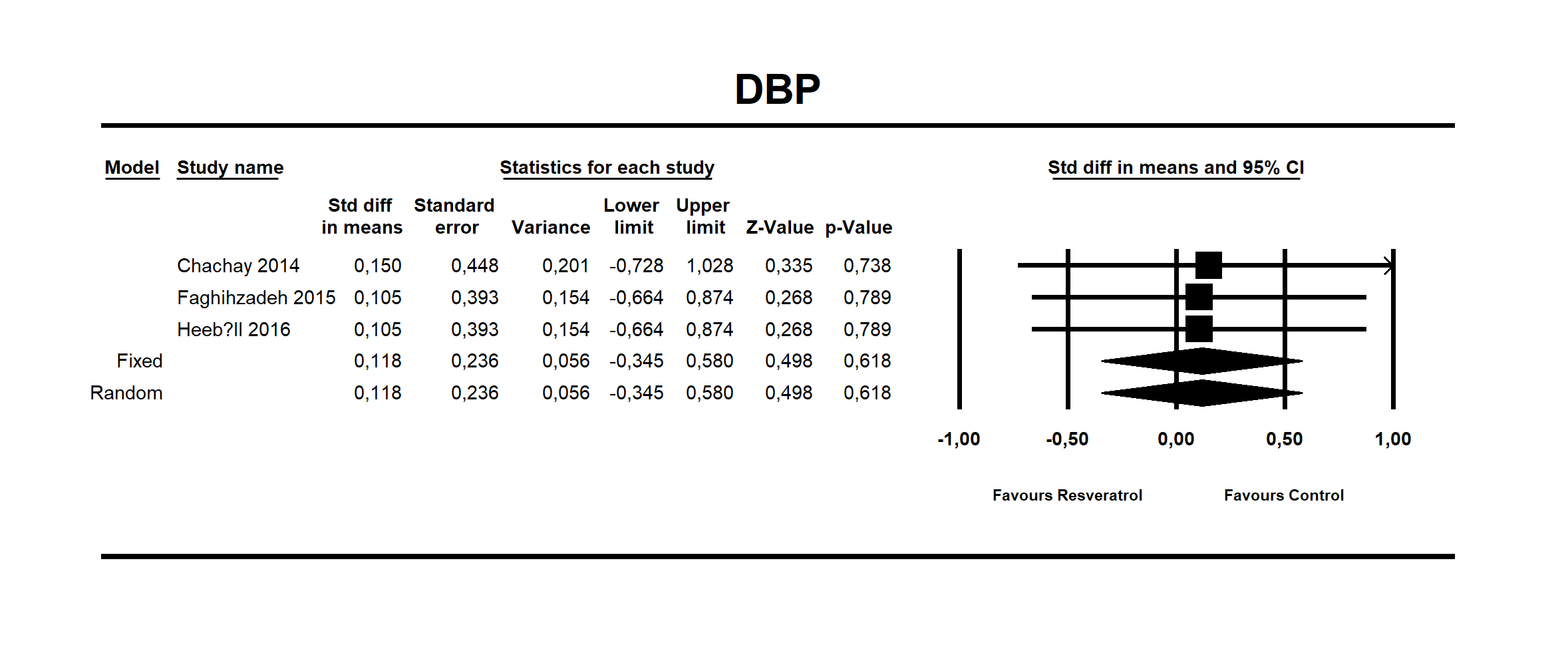
Figure 12.** An effect size, standardized mean difference, for DBP in persons taking RSV vs. controls (endpoint data). Q = 0.007, df(Q) = 2, *p* =0.996, I-squared = 0.0.


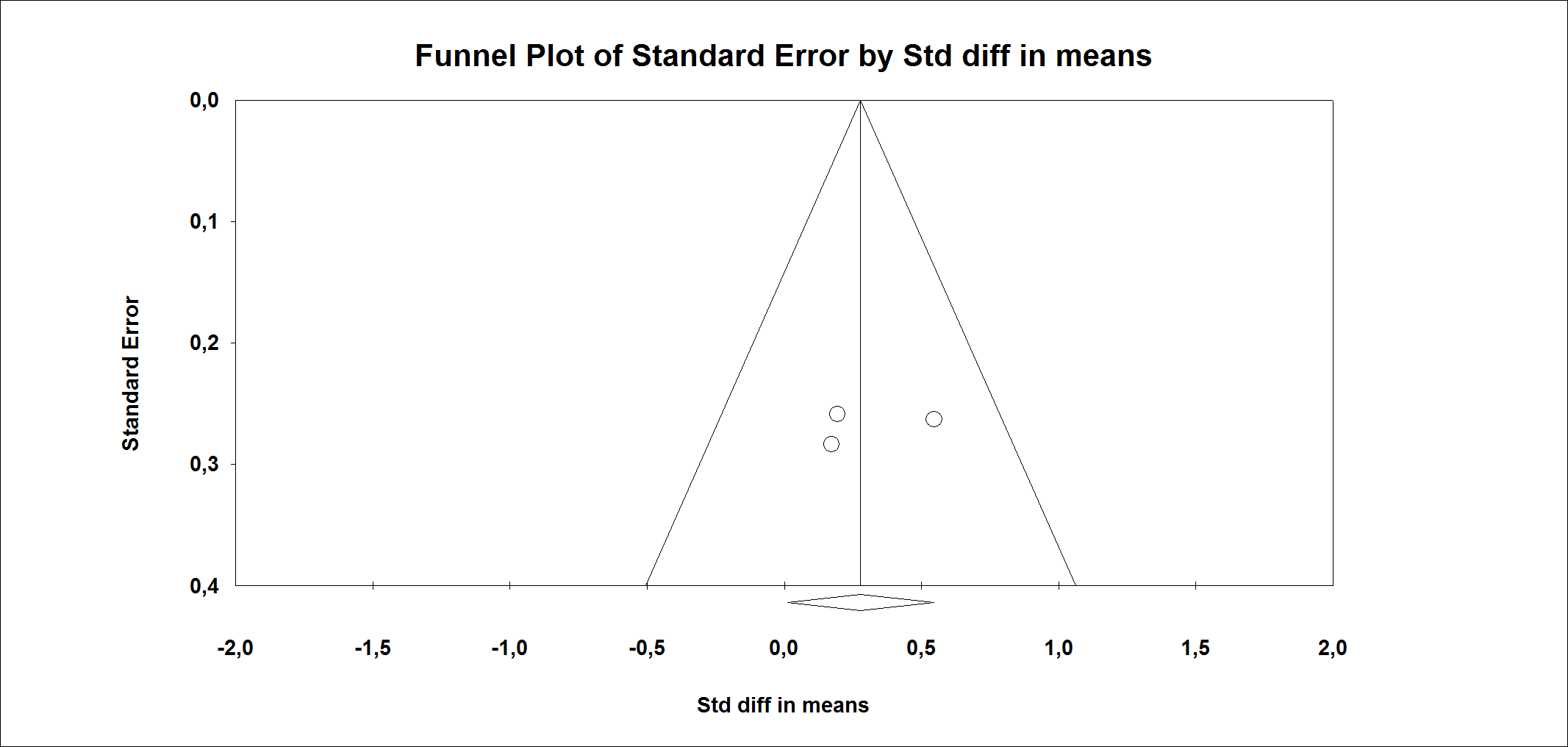
.**Figure 13.** Funnel plot for the effect of resveratrol on the level of ALT in patients with NAFLD in present meta-analysis.


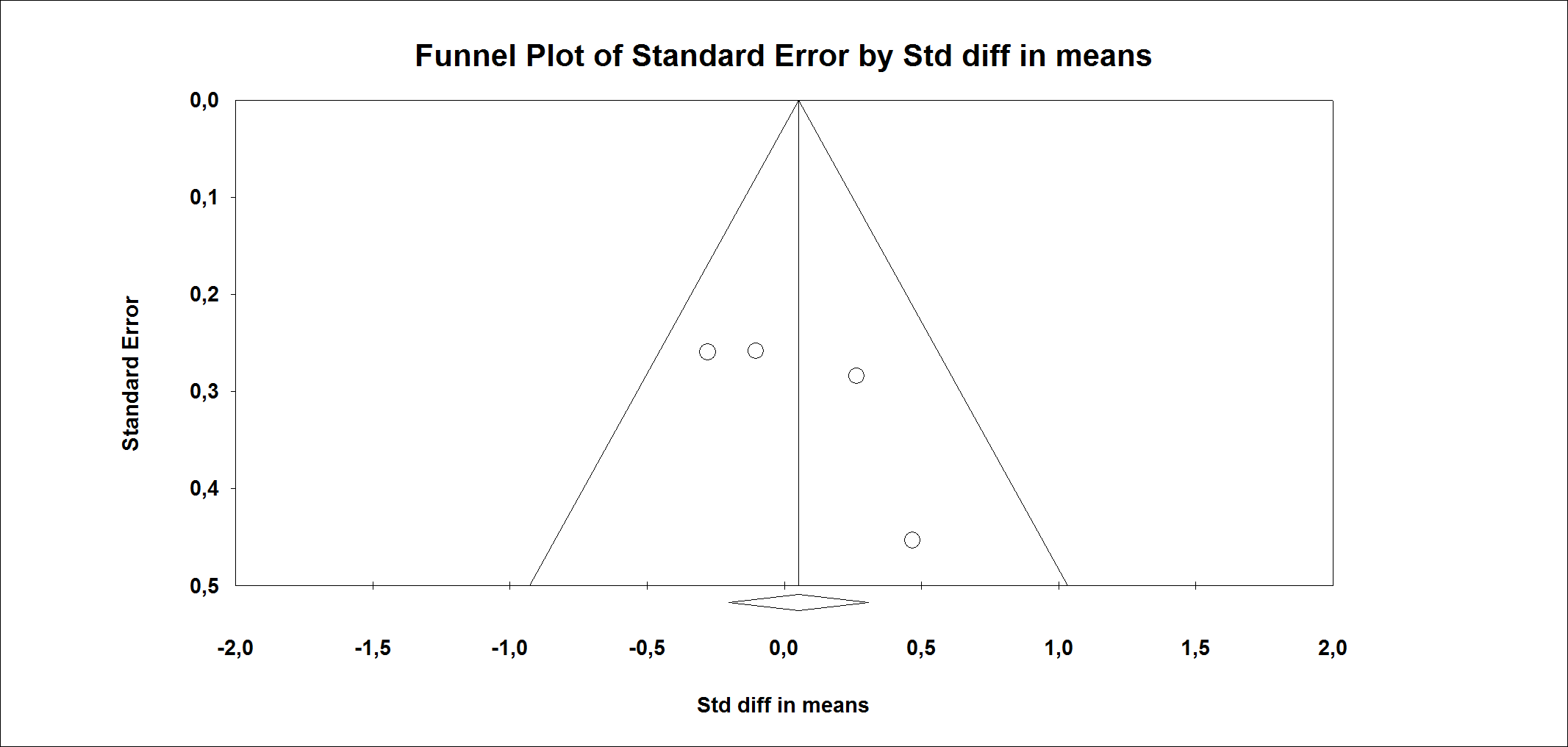


**Figure 14.** Funnel plot for the effect of resveratrol on the level of AST in patients with NAFLD in present meta-analysis.


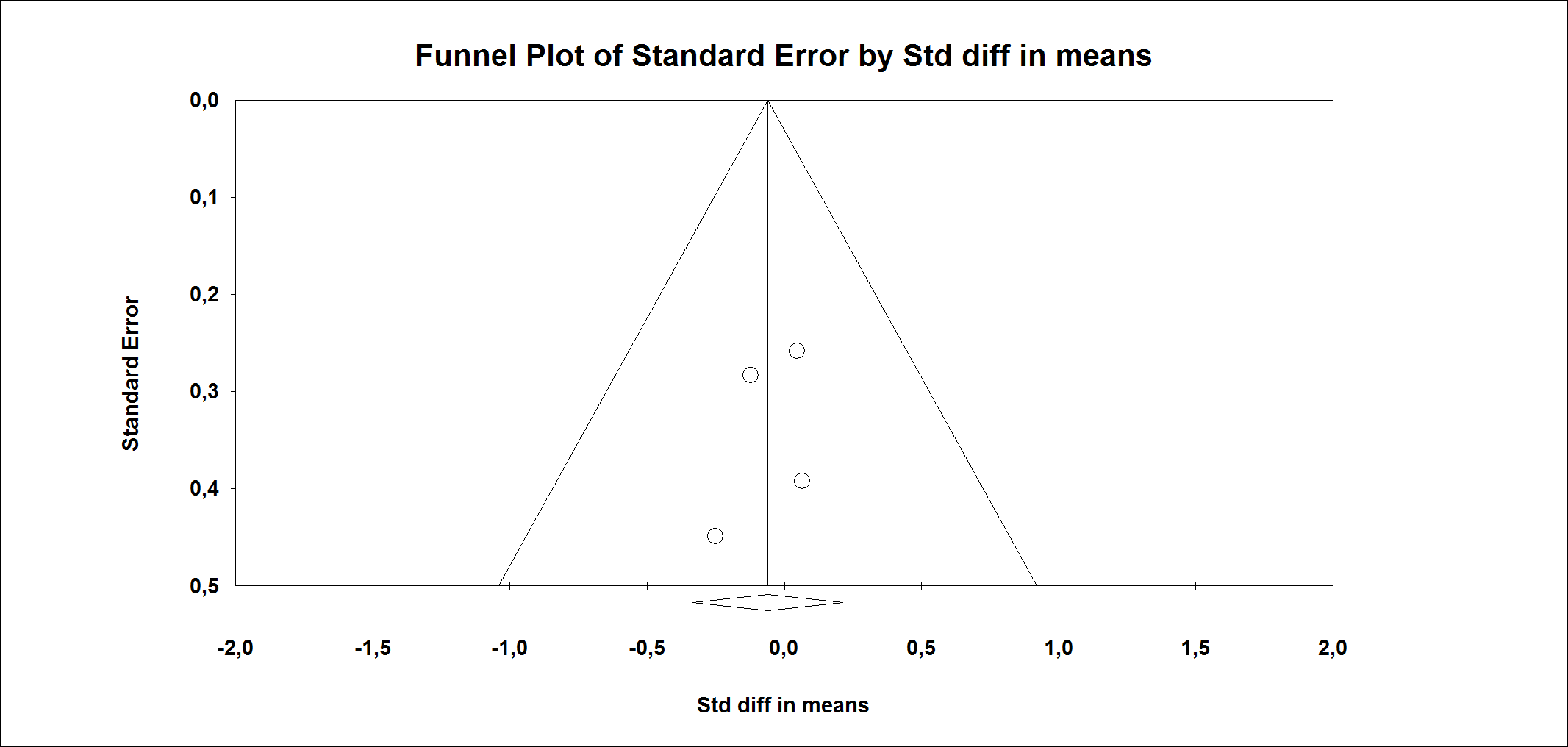


**Figure 15.** Funnel plot for the effect of resveratrol on the body weight in patients with NAFLD in present meta-analysis.


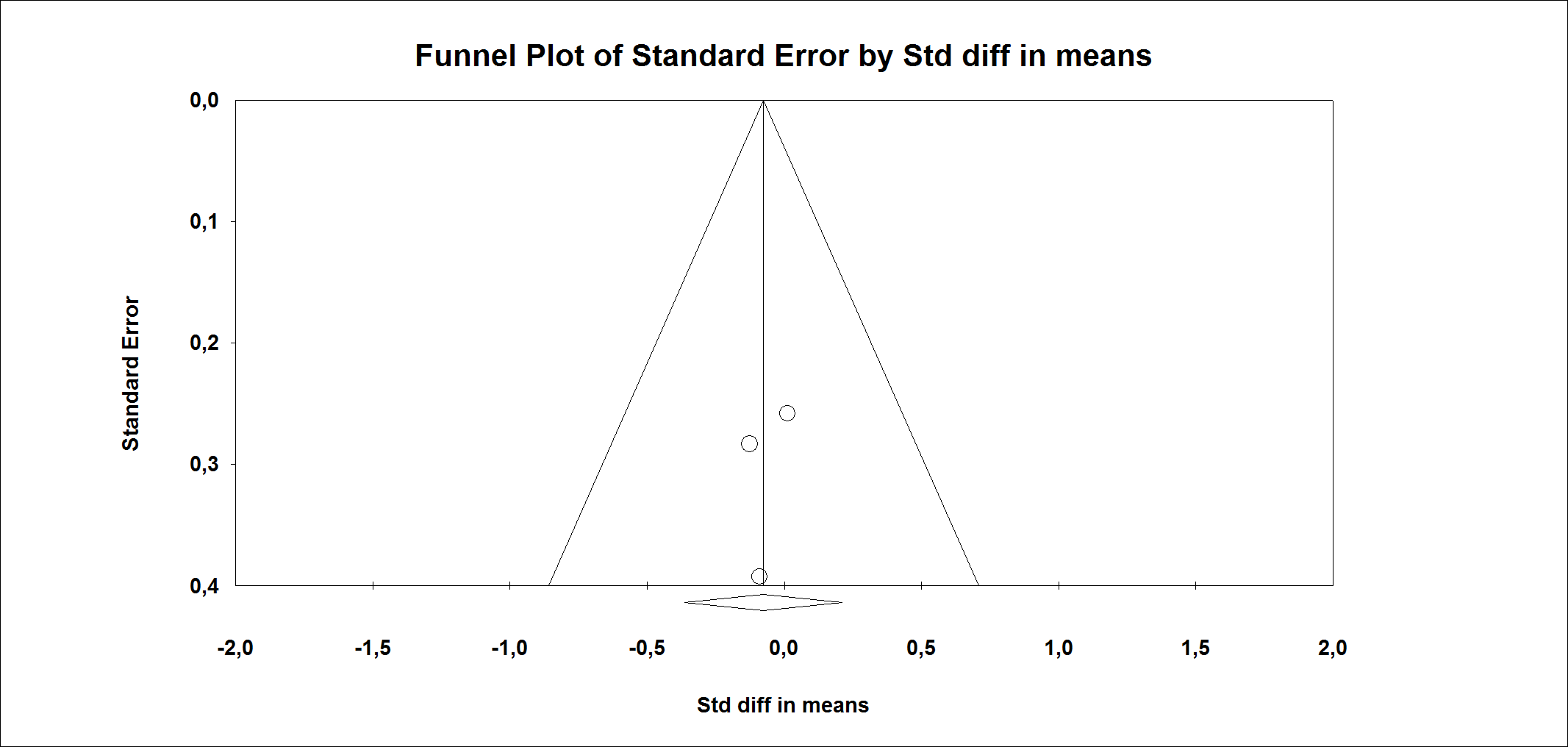


**Figure 16.** Funnel plot for the effect of resveratrol on the BMI in patients with NAFLD in present meta-analysis.


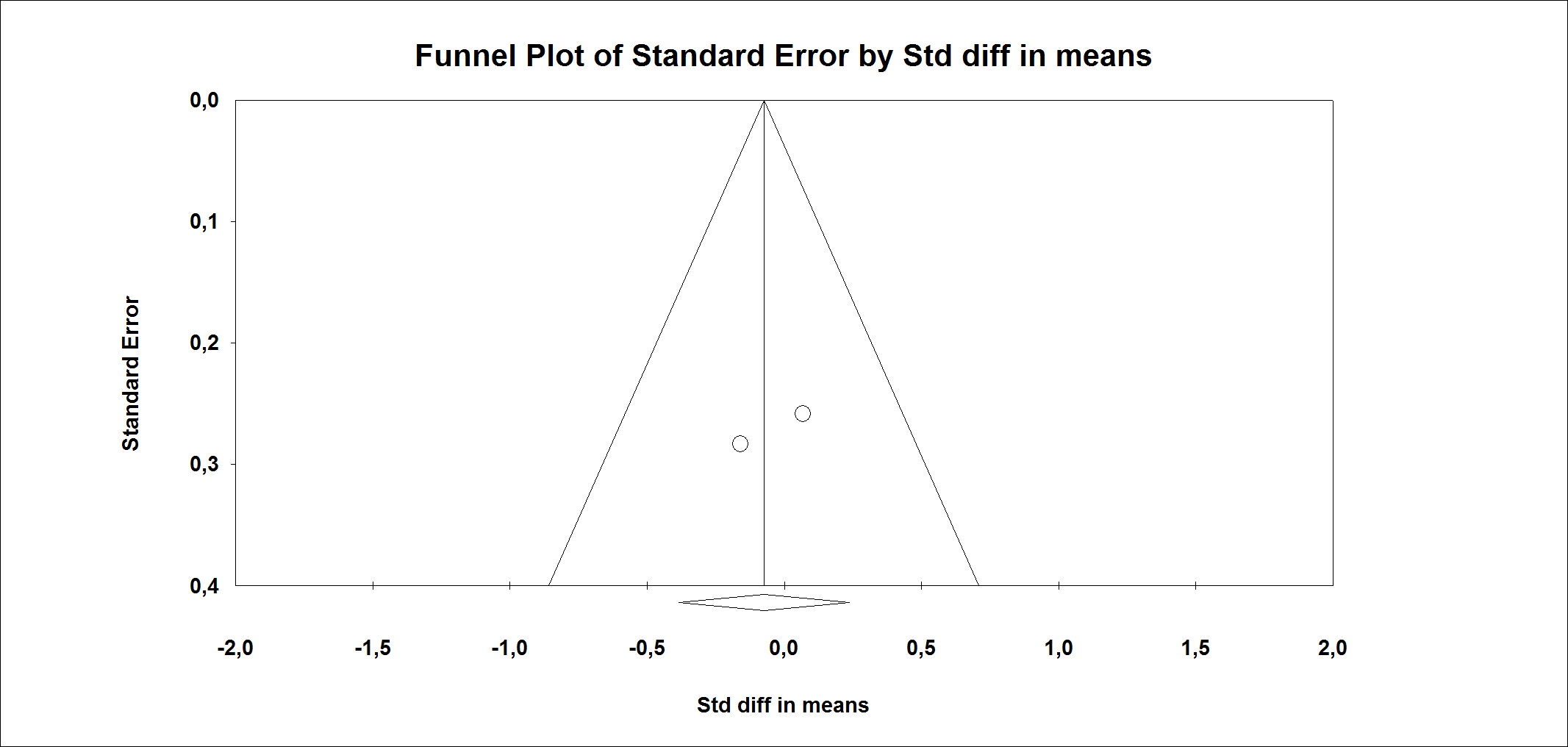


**Figure 17.** Funnel plot for the effect of resveratrol on the WC in patients with NAFLD in present meta-analysis.


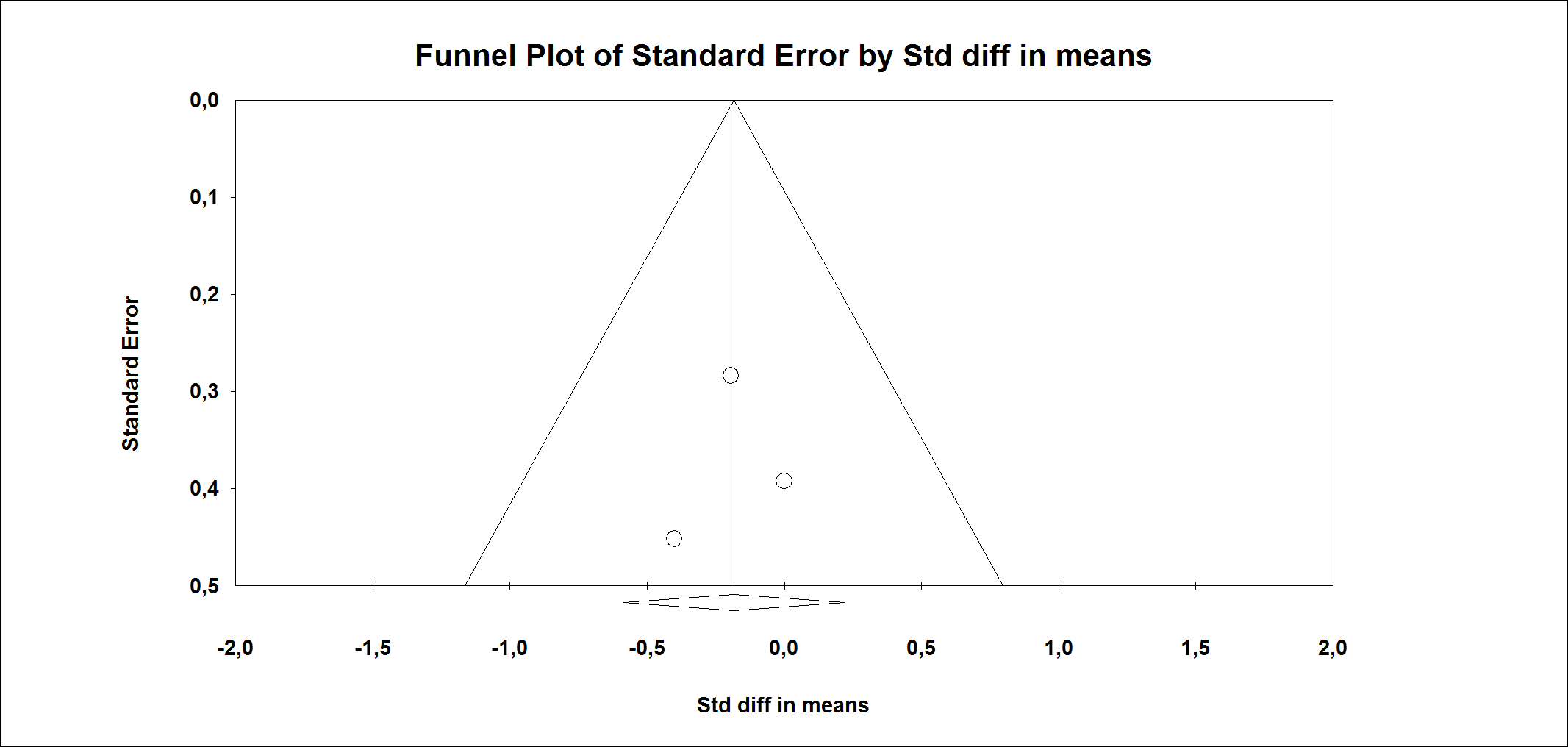


**Figure 18.** Funnel plot for the effect of resveratrol on the level of glucose in patients with NAFLD in present meta-analysis.


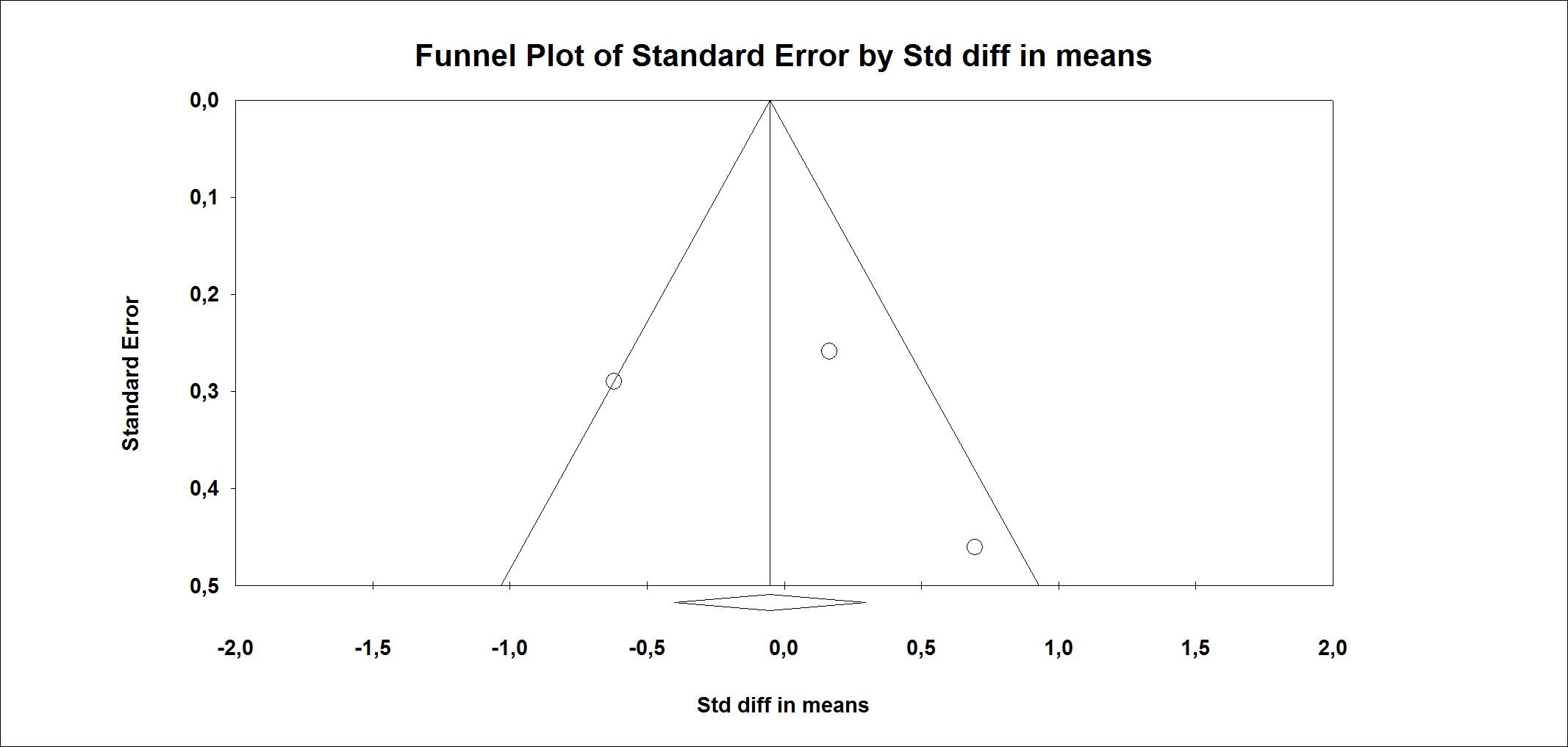


**Figure 19.** Funnel plot for the effect of resveratrol on the level of TC in patients with NAFLD in present meta-analysis.


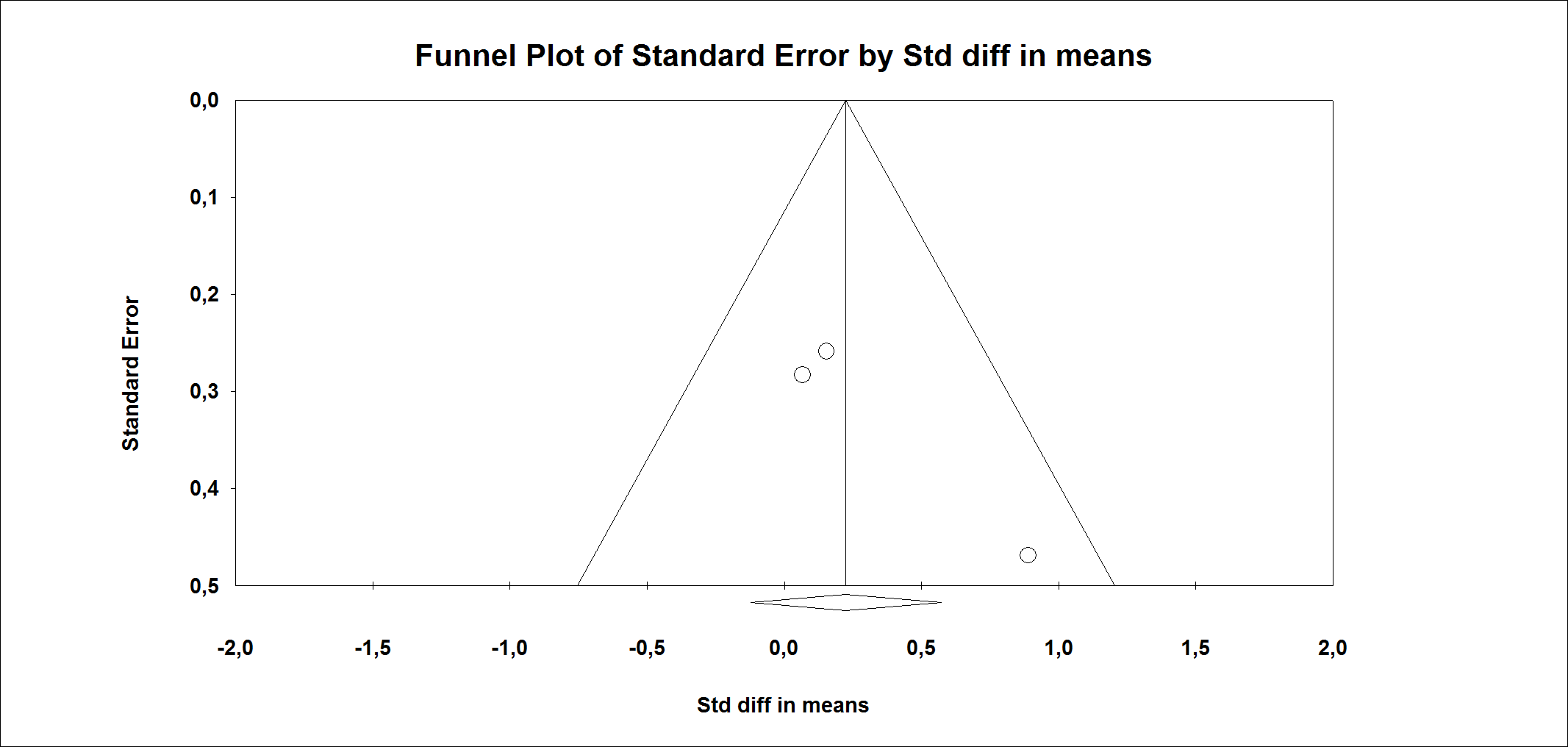


**Figure 20.** Funnel plot for the effect of resveratrol on the level of LDL in patients with NAFLD in present meta-analysis.


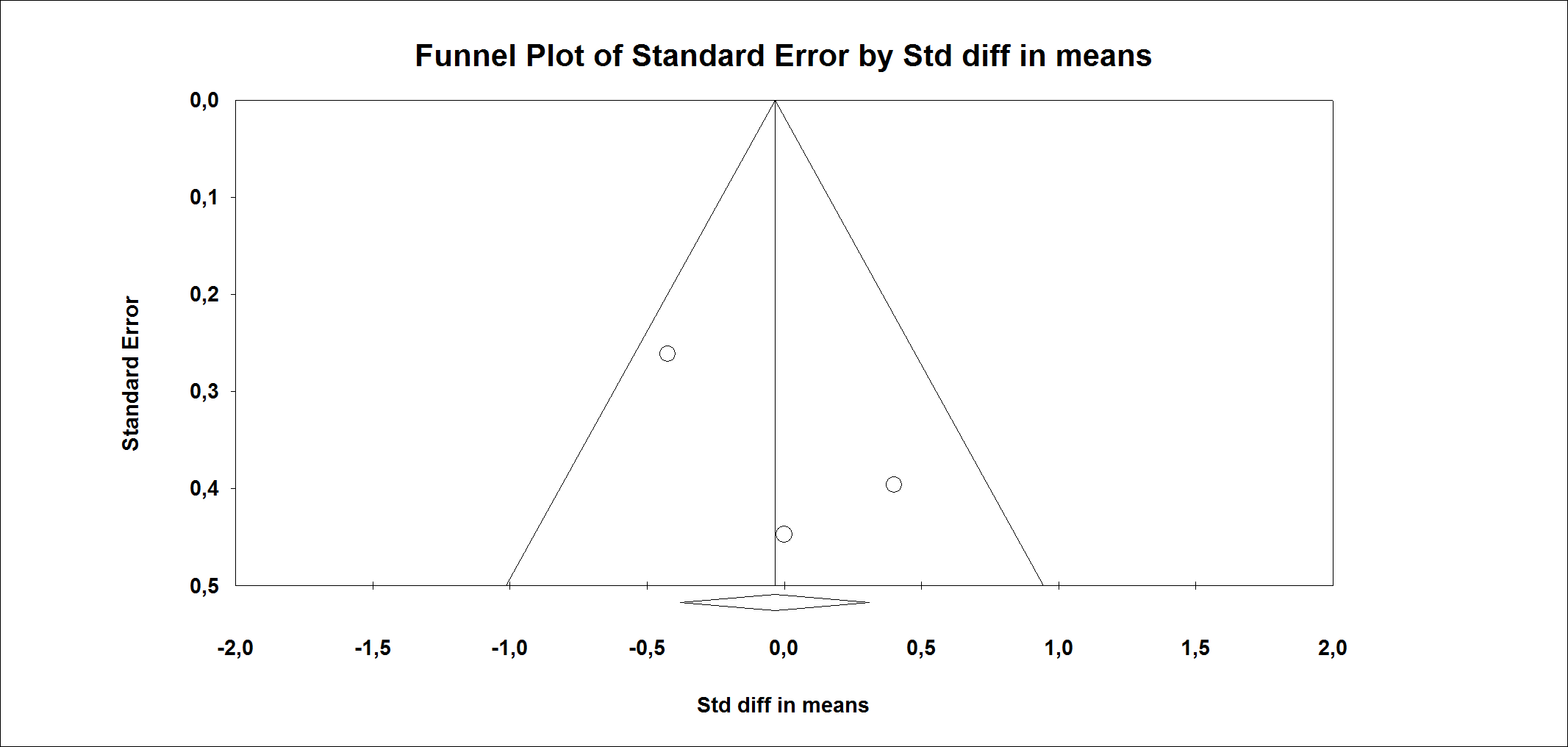


**Figure 21.** Funnel plot for the effect of resveratrol on the level of SBP in patients with NAFLD in present meta-analysis.


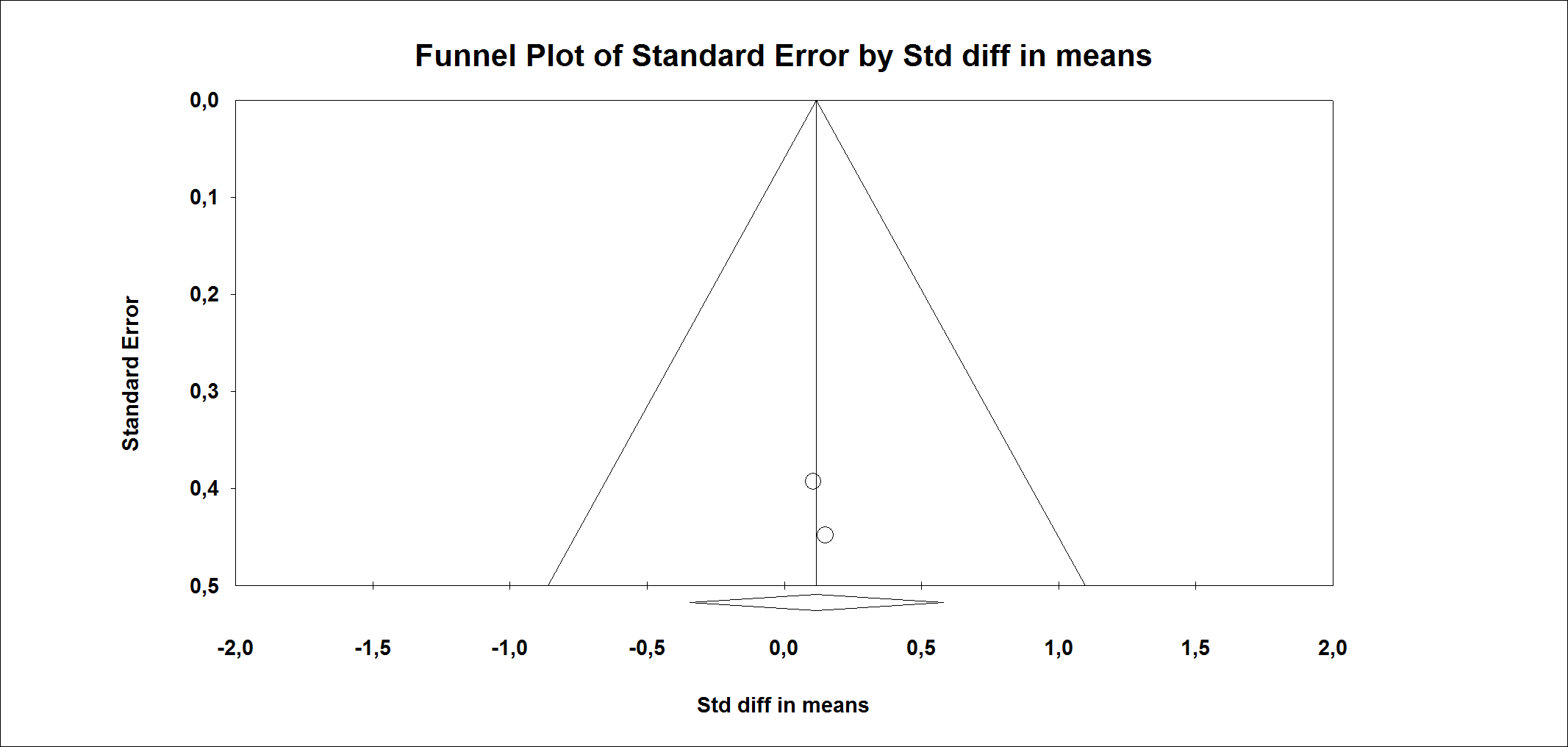


**Figure 22.** Funnel plot for the effect of resveratrol on the level of DBP in patients with NAFLD in present meta-analysis.
