# Supplementary material for: Effects of Resveratrol Supplementation in Patients with Non-Alcoholic Fatty Liver Disease—A Meta-Analysis
Source: Nutrients. 2020 Aug 13;12(8):2435. doi: 10.3390/nu12082435 (PMC7469003; doi:10.3390/nu12082435)
Supplement: Supplementary file 1 [file nutrients-12-02435-s001.zip › Supplementary_Table S1.docx]

**Table S1.** Additional parameters related to NAFLD that were not included in the meta-analysis.

| **REFERENCE** | **TREATMENT GROUP** | | **CONTROL GROUP** | | | | **TREATMENT GROUP** | | **CONTROL GROUP** | | | |
| --- | --- | --- | --- | --- | --- | --- | --- | --- | --- | --- | --- | --- |
|  | **MEAN** | **SD** | **n** | **MEAN** | **SD** | **n** | **MEAN** | **SD** | **n** | **MEAN** | **SD** | **n** |
|  | **ALT** | | | | | | | | | | | |
| Chachay et al. | 45 | 41-47 | 10 | 40 | 38-87 | 10 | 63 | 57-83 | 10 | 48 | 39-98 | 10 |
| Heeboll et al. | 104 | 56-225 | 13 | 86 | 28-235 | 13 | 74 | 35-179 | 13 | 72 | 27-160 | 13 |
|  | **AST** | | | | | | | | | | | |
| Heeboll et al. | 50 | 56-235 | 13 | 45 | 25-111 | 13 | 37 | 25-100 | 13 | 41 | 20-98 | 13 |
|  | **BMI** | | | | | | | | | | | |
| Chachay et al. | 31.8 | 30.2-37 | 10 | 31.2 | 27.4-39.3 | 10 | 37 | 30-37.4 | 10 | 31.1 | 28.1-40 | 10 |
|  | **Insulin** | | | | | | | | | | | |
| Asghari et al. 2018a | 43.61 | 1.38-240.97 | 30 | 40.34 | 1.38-142.36 | 30 | 34.37 | 1.38-229.86 | 30 | 43.26 | 1.38-472.22 | 30 |
| Chachay et al. | 83.33 | 55.55-173.61 | 10 | 69.44 | 55.55-97.22 | 10 | 104.16 | 55.55-180.55 | 10 | 90.27 | 62.5-111.11 | 10 |
|  | **LDL** | | | | | | | | | | | |
| Heeboll et al. | 3.2 | 1.8-6.3 | 13 | 2.4 | 1.7-7.4 | 13 | 3.4 | 1.8-6.3 | 13 | 2.8 | 1.5-5.6 | 13 |
|  | **DBP** | | | | | | | | | | | |
| Chen et al. | 80.6 | 8.76 | 30 | 84.5 | 14.4 | 30 | 79.4 | 12 | 30 | 85.13 | 10.8 | 30 |
|  | **HDL** | | | | | | | | | | | |
| Chachay et al. | 1 | 0.9-1.1 | 10 | 0.9 | 0.8-0.9 | 10 | 1 | 0.9-1.1 | 10 | 0.9 | 0.7-1.1 | 10 |
| Heeboll et al. | 1 | 0.72-1.9 | 13 | 1.2 | 0.8-1.7 | 13 | 1 | 0.82-1.6 | 13 | 1.1 | 0.83-1.9 | 13 |
|  | **TAG** | | | | | | | | | | | |
| Chachay et al. | 1.3 | 1.2-1.7 | 10 | 1.6 | 0.8-1.8 | 10 | 1.7 | 0.8-1.9 | 10 | 1.3 | 1.0-2.0 | 10 |
